# Ligand and structure-based toxicological assessment of (thio)semicarbazones on cholinesterases

**DOI:** 10.1007/s10822-025-00746-6

**Published:** 2026-01-08

**Authors:** Damião Sampaio de Sousa, Akenaton Onassis Cardoso Viana Gomes, Caio Henrique Alexandre Roberto, Anthony Barbosa Belarmino, Francisco Rogênio da Silva Mendes, Márcia Machado Marinho, Pedro de Lima-Neto, Gabrielle Silva Marinho

**Affiliations:** 1 Postgraduate Program in Natural Sciences, State University of Ceará, Fortaleza, CE Brazil; 2 Postgraduate Program in Biotechnology, State University of Ceará, Fortaleza, CE Brazil; 3 Natural Resources Bioprospecting and Monitoring Laboratory, Fortaleza, CE Brazil; 4 Department of Analytical and Physical Chemistry, Federal University of Ceará, Fortaleza, Ceará Brazil; 5 Faculty of Education, Sciences and Letters of Iguatu, Iguatu, CE Brazil

**Keywords:** Acetilcolinesterase, Toxicology, Computational techniques, Latency

## Abstract

Thiosemicarbazones (TSCBZ) are promising insecticidal compounds, but their potential neurotoxicity remains unclear. This study aimed to evaluate the toxicity and cholinesterase inhibition of six substituted TSCBZ derivatives using ligand- and structure-based computational approaches. Electronic property analysis revealed that bromine substitution enhances electrophilicity, while sulfur (in TSCBZ1–3) and nitrogen (in TSCBZ4–6) are the most nucleophilic sites. Toxicity prediction indicated that TSCBZ1, 4, and 6 may induce acute and chronic effects in aquatic organisms. Molecular docking showed that TSCBZ1 and TSCBZ4 exhibit higher affinity for acetylcholinesterase than galantamine, suggesting potential selective inhibition. These findings provide novel insights into the structure–toxicity relationship of TSCBZ and their environmental safety profile.

**Supplementary Information:**

The online version contains supplementary material available at 10.1007/s10822-025-00746-6.

## Introduction

Thiosemicarbazones have versatile biological properties and the ability to coordinate metal ions, reacting with the ions by bonding through thiocarbonyl sulfur and azomethine nitrogen atoms, and have the general formula R_1_R_2_CH = N-NH-(C = S)-NH_2_ (Hernández et al. 2020; Lira et al., 2022). This class of molecules has shown promise in research into new drug candidates, due to their broad spectrum of action (Soares et al. 2017). Among their biological activities are: antitumor, antiparasitic, antifungal, antiviral and antibacterial (Alam et al. 2023).

Semicarbazones and thiosemicarbazones are important compounds in the development of analogs that have an insecticidal action (De Siqueira et al. 2019). In addition, their derivatives usually show good selectivity and low toxicity to non-target organisms (Aly et al. 2010; Silva et al. 2013). These derivatives provide a representative set for exploring structure–activity relationships relevant to cholinesterase inhibition, a key mechanism underlying neurotoxicity in aquatic organisms. Moreover, the selection allows comparison with previously studied compounds, providing insight into how specific structural modifications affect predicted toxicity and enzyme selectivity (Langat et al. 2024; Khan et al. 2023). This rationale ensures that our computational analysis addresses compounds of both biological and ecological relevance (Naseem et al. 2024; Cheng et al. 2021).

Although thiosemicarbazones have a low toxic effect on non-target organisms, studies are needed to target organisms, there is a need for studies to assess their environmental impact, since pesticides when sprayed can can contaminate rivers and lakes, affecting living beings in that environment. Among the possible harmful effects is neurotoxicity, caused by inhibition of the enzymes acetylcholinesterase (AChE) and butyrylcholinesterase (BChE). This inhibition results in the accumulation of the neurotransmitter acetylcholine (ACh), leading to overstimulation of cholinergic pathways and potential neurological, behavioral, and cognitive impairments (Čadež et al. 2021; Ortiz-Delgado et al. 2021).

In vivo studies have shown that exposure to organophosphate insecticides induces AChE inhibition in albino rats, causing neuromuscular dysfunction and, consequently, latency (Mohan et al. 2024). ACh neurotransmitters play a central role in critical physiological processes such as attention, learning, memory, stress response, sleep and sensory information (Francis et al. 2012; Wright 2020). Meanwhile, butyrylcholine (BCh) is a substrate of BChE, which hydrolyzes ACh and plays a role in neuronal development (Kinchen et al., 2021; Yamamoto and Momonoki 2021).

The aim of this study is to investigate the effects of structural substitutions of thiosemicarbazones (thiophene and furan) on toxicity in aquatic organisms. Computational approaches will be used to analyze the relationship between chemical structure and biological activity. In addition, the study incorporates environmental monitoring studies, the structural characterization of the molecules and the evaluation of the stability of the interactions between ligands and enzymes, as well as the biochemical interactions related to cholinergic modulation.

## Materials and methods

The molecules were selected from the study by Cheng et al. (2021), who synthesized semicarbazones and thiosemicarbazones, which showed insecticidal potential against *Leucania separata* and *Pieris rapae*, when compared to *beta*-cypermethrin.

**Fig. 1:**
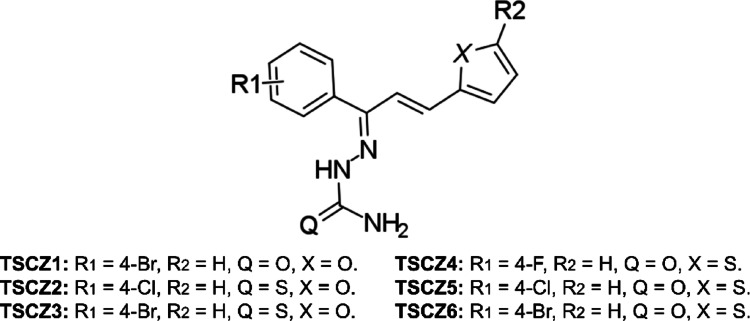
Two-dimensional of thiosemicarbazones *Source*: Adapted from Cheng et al. (2021)

Because of this, to complement the analyses, in silico quantum, protein targeting and ecotoxicological studies were carried out on the thiosemicarbazones (**2e**-TSCZ1; **2f**-TSCZ2; **2 g**-TSCZ3; **2i**-TSCZ4; 2j-TSCZ5; and 2k-TSCZ6) due to their similarity in structure and physicochemical properties, making it possible to assess their environmental impact.

### Density functional theory (DFT)

Geometry optimizations and electronic property calculations were performed using the DFT method with the B3LYP functional and the 6-311 + + G(d, p) basis set. This level of theory was chosen because it provides reliable accuracy for organic molecules containing sulfur and halogens while maintaining reasonable computational efficiency (Iczkowski and Margrave, 1961).

The geometry optimization data, frontier orbitals, global and local quantum descriptors, electronic functions, and Fukui condensates were obtained in the Gaussian 09 program using Becker’s three-parameter hybrid functional (Becker, 1993) and Lee, Yang and Parr correlation functionals (B3LYP) (Lee et al. 1988) with a Pople 6-311 + + G(d, p) basis set (Pople and Nesbet 1954). The structures were optimized in water (ε = 78.3553) using the Integral Equation Formalism - Polarizable Continuum Model (IEF-PCM) (Mennucci et al. 1997; Cances et al. 1997) for the implicit solvent solvation method.

The isodensities of the HOMO (Highest Occupied Molecular Orbital) and LUMO (Lowest Unoccupied Molecular Orbital) orbitals were plotted and, the energy values were used to calculate the energy gap (ΔE, Eq. 1 ) (Pearson 1963) Ionization Potential (I, Eq. 2) and Electronic Affinity (*EA*, Eq. 3) (Koopmans 1934), Electronegativity (χ, Eq. 4) (Chermette 1999; Iczkowsk and Margrave, 1961), Global Hardness (η, Eq. 5) (Janak 1978; Pearson 1987; Von-Szentpály 1991), Global Softness (S, Eq. 6) (Yang and Parr 1985), Electrophilicity Index (ω, Eq. 7)(Parr et al. 1999), and Nucleophilicity Index (ϵ, Eq. 8) (Chattaraj; Giri and Duley, 2011).

1 $$\:\varDelta\:E={E}_{LUMO}-\:{E}_{HOMO}$$

2 $$\:I=-\:{E}_{HOMO}$$

3 $$\:EA=-\:{E}_{LUMO}$$

4 $$\:\chi\:=(I+A)/2$$

5 $$\:\eta\:=(I-A)/2$$

6 $$\:S=1/\eta\:$$

7 $$\:\omega\:={\chi\:}^{2}/2\eta\:$$

8 $$\epsilon=1/\omega\:$$

9 $$\:{f}_{A}^{+}=\:{q}_{N}^{A}-\:{q}_{N+1}^{A}$$

10 $$\:{f}_{A}^{-}=\:{q}_{N-1}^{A}-\:{q}_{N}^{A}$$

11 $$\:\varDelta\:f=\:{f}^{+}-\:{f}^{-}$$

12 $$\:\varDelta\:\omega\:=\:\omega\:\varDelta\:f$$

Condensed Fukui functions were calculated using the Hirshfeld charge population analysis in two situations, one for susceptibility to nucleophilic attack (*f* ^+^, Eq. 9) (Morell et al. 2005) and the other for susceptibility to electrophilic attack (*f* ^-^, Eq. 10). Dual descriptor (Δ*f*, Eq. 11) was calculated concomitantly with the multiphilic descriptor (Δω, Eq. 12) (Padmanabhan et al. 2007) to estimate nucleophilic and electrophilic atomic centers simultaneously. If Δf and Δw < 0, the reactive site has a nucleophilic character. If Δf and Δw > 0, the reactive site is electrophilic. Molecular Electrostatic Potential (MEP) was calculated and plotted to investigate electronic density distribution in the different environments analyzed.

### Ecological structure activity relationship (ECOSAR^®^) and BCFBAF

The ECOSAR^®^ software is based on Quantitative structure-activity relationship (QSAR) models to predict the toxic effect of toxicants on aquatic species, using the octanol-water partition coefficient (log_Kow_) as the main parameter (Feng et al. 2024). The descriptor is calculated by dividing the molecule into smaller fragments that contribute to a final score that calculates the log_Kow_, and toxicity is measured in lethal (LC_50_) and effective (EC_50_) concentrations for acute effects and in chronic value (ChV) for chronic toxicity (Carpinteiro et al. 2017) (Table 1).

**Table 1:** Classification of acute and chronic toxicity ECOSAR^®^ *Source*: Adapted from Manonmani et al. (2021)

Classification	Acute toxicty	ChV* toxicity
Not harmful	LC_50_/EC_50_ > 100	ChV > 100
Harmful	10 < LC_50_/EC_50_ < 100	1 < ChV < 10
Toxic	1 < LC_50_/EC_50_ < 10	0.1 < ChV < 1
Very toxic	LC_50_/EC_50_ < 1	ChV < 0.1
Unit: mg/L^-1^		

*ChV: chronic

The following equations are applied to neutral organic compounds in the calculations that determine concentrations and toxic effects:

13 $$\:\text{log}{LC}_{50}=m\bullet\:\text{log}kow+b$$

14 $$\:{A}_{nar}\approx\:\frac{{C}_{nar}}{{C}_{s}}$$

15 $$ \log 1/HC_{{5aqcom}} = - 4.52 + 1.05 \cdot \log kow $$

16 $$\:ChV=\frac{\text{log}(NOEC\cdot\:LOEC)}{2}$$

Equation 13 refers to a linear regression between the log LC_50_ in mmol/L (acute toxicity) and the log_Kow_ (Ibrahim and Odedele 2018). However, Eq. 14 indicates the calculation of narcosis (acute effect) under conditions where the compound in the organism’s blood is thermodynamically stable with water, where A_nar_ informs the chemical activity as a narcotic, C_nar_ the concentration in water necessary to induce narcosis (after LC_50_) and C_s_ the solubility in water (Veith et al. 1983).

Equation 15 calculates the hazardous concentration for 5% of aquatic communities (HC_5aqcom_ in µmol/L) of narcotics at different trophic levels, such as plants, invertebrates, and vertebrates, using log_Kow_ (Finizio et al. 2020). Equation 16 consists of the geometric mean between the no-observed effect concentration (NOEC) and the lowest observed effect concentration (LOEC) (Roveri et al. 2020).

From this perspective, the BCFBAF^®^ prediction model is a software program that calculates bioconcentration (BCF) and bioaccumulation (BAF) factors in fish from three trophic levels using the Arnot-Gobas method (Arnot and Gobas 2003); this method considers factors such as biotransformation, growth dilution, and the elimination of xenobiotics through the surface of the gills and feces (Kropf et al. 2020).

17 $$\:ChV=\frac{\text{log}(NOEC\cdot\:LOEC)}{2}$$

18 $$\:BCF=\left(1-{L}_{B}\right)+\left(\frac{{K}_{1}\cdot\:\varnothing\:}{{K}_{2}+{K}_{E}+{K}_{G}+{K}_{M}}\right)$$

Equations 17 and 18 show how BCF and BAF are predicted, where 1-_LB_ indicates the components of the organism, K_1_, K_2_, K_E_, K_G_ and K_M_ are constants that show the absorption of compounds through respiration and diet, as well as elimination through respiration, fecal, growth dilution and metabolic transformation, respectively, ϕ shows the concentration of the substance that dissolves and can permeate the respiratory membranes, β shows the biomagnification factor and τ denotes trophic dilution (Arnot and Gobas 2003).

In addition, the JANUS^®^ tool is often used to analyze PBT (Persistence, Bioaccumulation, and Toxicity) and CMR (Carcinogenicity, Mutagenicity, and Reprotoxicity) endpoints (Toma et al. 2021). In this study, JANUS^®^ was used to assess the environmental distribution of thiosemicarbazones, predicting persistence in soil, water, and sediment compartments (Lombardo et al. 2022).

### Statistical analysis

The GraphPad Prism^®^ software was used to evaluate toxicity and effects, incorporating the Shapiro-Wilk normality test with p-value ≥ 0.05, as well as analysis of variance (One and Two-Way-ANOVA), using the Tukey post-hoc test for multiple comparisons to identify statistically significant differences (p-value < 0.05) for acute/chronic concentrations between the aquatic species of thiosemicarbazone derivatives.

### CNS MPO and screening of target binding

Latency (narcosis or baseline toxicity) consists of the minimum toxicity that a chemical product (called toxicant) can cause based on its lipophilicity from the perspective of bioaccumulation in the lipid compartments of organisms. In this way, neurobehavioral toxicity syndromes (BTS) enable an underlying approach indicated by the inhibition of AChE or BChE characterized by a high incidence of convulsions, anxiolytic, scoliosis, and hemorrhage in the brain área (Claeys et al. 2013; Bradbury et al. 1989).

In addition, lethal effects on the identification and characterization of baseline toxicity at the molecular level were initially predicted using Pfizer’s CNS Multiparameter Optimization (CNS MPO) algorithm (Wager et al. 2010), which employs quantitative drug similarity, as described in Eq. 19.

19 $$\:BCF=\left(1-{L}_{B}\right)+\left(\frac{{K}_{1}\cdot\:\varnothing\:}{{K}_{2}+{K}_{E}+{K}_{G}+{K}_{M}}\right)$$

where *w* is the weighting factor (0 to 1) assigned to each physicochemical property *k* that falls within (x_k_ < x_a_) or outside the function (x_b_ < x_k_) of ideal desirability (T (*x*), the attributes include intrinsic lipophilicity (logP adapted for log_Kow_ ≤ 3), polar surface area (40 < TPSA ≤ 90Å2), pH buffer lipophilicity (logD ≤ 2), acidity/basicity (pka ≤ 8.0), molecular weight (MW ≤ 500 g/mol) and H-bond donors (HBD ≤ 1), thus, the score for CNS desirability is calculated by adding up the values of six (6) items ranging from 0 (low CNS desirability) to 6 (good CNS desirability) (Marinho et al. 2024).

The target prediction structure was made possible by drawing the two-dimensional TSCBZ1-6 structures using the academic license software MarvinSketch^®^ version 24.1.2 (ChemAxon) and converting them into SMILES (Simplified Molecular-Input Line-Entry System). Subsequently, the SMILES notations of the compounds were directed to the SwissTargetPrediction (http://www.swisstargetprediction.ch/) webserver (Daina et al. 2019), which performs 3D/2D similarity analysis with bioactive compounds against biological enzymes expressed in *Mus musculus*, *Homo sapiens*, *and Rattus norvegicus* organisms, as described in Eq. 20.

20 $$\:\text{d}=\sum\:_{\text{s}=1}^{18}\left|\text{x}\text{s}-\text{y}\text{s}\right|$$

The 3D similarity can be determined by the Manhattan distance (*d*) applied to compare two different molecules described by the vectors x and y in their respective conformations (*s*). In this way, the comparison is implemented between the 20 most stable conformations of molecules *x* and *y* (*s* = 18) in which a training set is added with ChEMBL data, including inhibitory concentration (IC_50_) and inhibition constant (K_i_) values (Gfeller et al. 2014). To assess the similarity of bioactive compounds for the biological target AChE, we analyzed each ligand by comparing it with structurally related known bioactive molecules, selecting the most relevant candidate ligands for detailed evaluation.

### Molecular Docking

The methodological path of molecular docking is shown Table 2, which shows the software’s and parameters used in this study.

**Table 2:** Softwares and parameters applied in the study

Softwares	Version	Link	Setting	Fuction	Citation
MarvinSketch^®^	24.1.2	https://chemaxon.com/marvin	pH = 7.4 logP (kow) ≤ 3 CNS MPO score	Two-dimensional ilustration	Giménez et al. (2010)
Avogadro^®^	1.2.0	https://avogadro.cc/	MMFF94 *Steepest descent* (50 interaction)	Structural optimization	Snyder and Kucukkal (2021)
AutodockTools^®^	1.5.6	https://autodock.scripps.edu/	∆G = − 6.0 kcal/mol^−1^ RMSD ≤2	Molecular docking simulations	Morris et al. (2008)
RCSB PDB^©^	webserver	https://www.rcsb.org/	PDB: 4EY6 (AChE with GNT) PDB: 7AIY (BChE with 8U2)	Acess and tools for exploration, visualization and analysis of macromolecules	Kouranov et al. (2006)
UCSF Chimera^®^	1.18	https://www.cgl.ucsf.edu/chimera/	Removal of residues and addition of polar hydrogens	Visualization and analysis of molecular structures	Pettersen et al. (2004)
Discovery Studio Visualizer^®^	21.1	https://discover.3ds.com/discovery-studio-visualizer-download	Visualization of the protein-ligand complex	Visualization of small macromolecules	Pawar and Rohane (2021)
Protein-ligand Interaction Profile^©^	webserver	https://plip-tool.biotec.tu-dresden.de/plip-web/plip/index	Interactions between protein-ligands ≥ 5Å	Identification of interactions between protein-ligands	Salentin et al. (2015)
Pymol^®^	4.6	https://www.pymol.org/	Visualization of interactions between ligand and amino acid residues	Visualization system molecular	Yuan et al. (2017)

logKow (Octanol-water partition coefficient), CNS MPO (Central Nervous System Multiparameter Optimization), MMFF94 (Merck Molecular Force Field), ∆G (Affinity energy), RMSD (Root Mean Square Deviation), GNT (galantamine) and 8U2 (2-｛1-[4-(12-amino-3-chloro-6,7,10,11-tetrahydro-7,11-methanocycloocta[b]quinolin-9-yl)butyl]-1H-1,2,3-triazol-4-yl｝-N-[4-hydroxy-3-mehoxybenzyl]acetamide)

### Molecular dynamics

Molecular dynamics (MD) simulations were carried out using the GROMACS 2022.6 software (Bauer et al. 2023), in which the CHARMM36m force field (Huang et al. 2017) was applied to the protein. The force field of the ligands was determined using the SwissParam tool. Then, the ligand-protein complexes were solvated with water molecules (H_2_O) using the TIP3P model (transferable intermolecular potential with 3 points) (Mark and Nilsson 2001; Neria et al. 1996).

The systems were neutralized with a physiological concentration of 0.15 mol/L, containing 145 Na^+^ ions and 147 Cl^-^ ions. Then, energy minimization was deliberated by applying the steepest descent method, configured to stop the process when the force was less than 10.0 kJ/mol. After minimization, the system was subjected to thermal equilibration in two stages: first, with the number of particles, volume, and temperature constant (NVT), and then, with the number of particles, pressure, and temperature controlled simultaneously (NPT). Once equilibration was complete, molecular dynamics (MD) simulations were carried out at 310 K and 1.0 bar for 200 ns, with three independent simulations carried out for each system.

21 $$\:RMSD=\sqrt{\frac{1}{N}{\sum\:}_{i=1}^{N}{\left({r}_{i2}-{r}_{i1}\right)}^{2}}$$

22 $$\:RMSF=\sqrt{\frac{1}{N}{\sum\:}_{t=1}^{N}{\left({r}_{i}\left(t\right)-{\{r}_{i}\}\right)}^{2}}$$

23 $$\:{G}_{bind}={E}_{vdW}+{E}_{ele}+{G}_{GB}+{G}_{SA}-T\varDelta\:S$$

The results were analyzed by calculating Root Mean Square Deviation (RMSD) (Eq. 21), Root Mean Square Fluctuation (RMSF) (Eq. 22), Molecular Mechanics of Generalized Born Surface Area (MM/GBSA) (Eq. 23), and hydrogen bond frequencies. The stability of the complexes over time was assessed by analyzing the structural coordinates extracted from the molecular dynamics (MD) trajectories.

Where *N* is the total number of atoms, ri^2^ is the atom’s position in the last calculated frame, and ri^1^ is the position of the reference atom (initial position). Measuring the average deviation of atomic positions between two frames is possible.

Where *N* is the total number of frames in the simulation, ri(*t*) is the atom’s position at time t, and {r*i*} is the average position of atom *i* in the *N* simulated frames.

Where the first two terms are the van der Waals interactions (E_vdW_) and electrostatic energies (E_ele_), the term (G_GB_) represents the polar contributions and (G_SA_) are the nonpolar contributions to the solvation free energies, the last term is the absolute temperature (T), multiplied by the entropy variation (ΔS), being estimated by a normal mode analyzing the vibrational frequencies (Desheng et al. 2011; Genheden and Ryde 2015).

Overall, the molecular dynamics (MD) simulations, the energy minimization and equilibration thresholds were chosen to ensure numerical stability, proper convergence, and physically meaningful trajectories. The cutoff distances, time step, and equilibration period were based on standard protocols for enzyme–ligand complexes, ensuring accurate sampling of conformational space and stable temperature and pressure profiles throughout the simulation. These settings are consistent with previously validated MD studies of cholinesterase–ligand systems.

## Results

### DFT calculations

The investigation by theoretical model was carried out on synthetic derivatives containing in the structural skeleton the following groups: thiosemicarbazones (TSCZ1-3) or semicarbazones (TSCZ4-6), unsaturated linear chain, furan ring (TSCZ1-3) or thiophene (TSCZ4-6) and aromatic ring substituted in the para position with the F (1), Cl (2) or Br (3) atoms. This research was conducted to evaluate the structural, electronic, and chemical reactivity properties of the molecules of interest. The geometric data was obtained by DFT calculations at the B3LYP/6-311 + + G(d, p) level of theory, considering the water environment to simulate the biological analysis environment.

The frequency data showed that the optimized structures are at a minimum on the potential energy surface. The geometrically optimized structures are shown in Fig. S1, exhibiting low symmetry characterized by the C1 point group. The benzene group is positioned almost orthogonally to the linear chain in all the structures to minimize repulsion between the hydrogens of both groups.

#### Frontier molecular orbitals (FMO)

In the study of quantum chemistry, HOMO and LUMO frontier orbitals are essential parameters for understanding electronic properties, stability, and chemical reactivity and can also be used to explain intermolecular interactions and charge transfer with proteins of interest (Maidur et al. 2017).

**Fig. 2:**
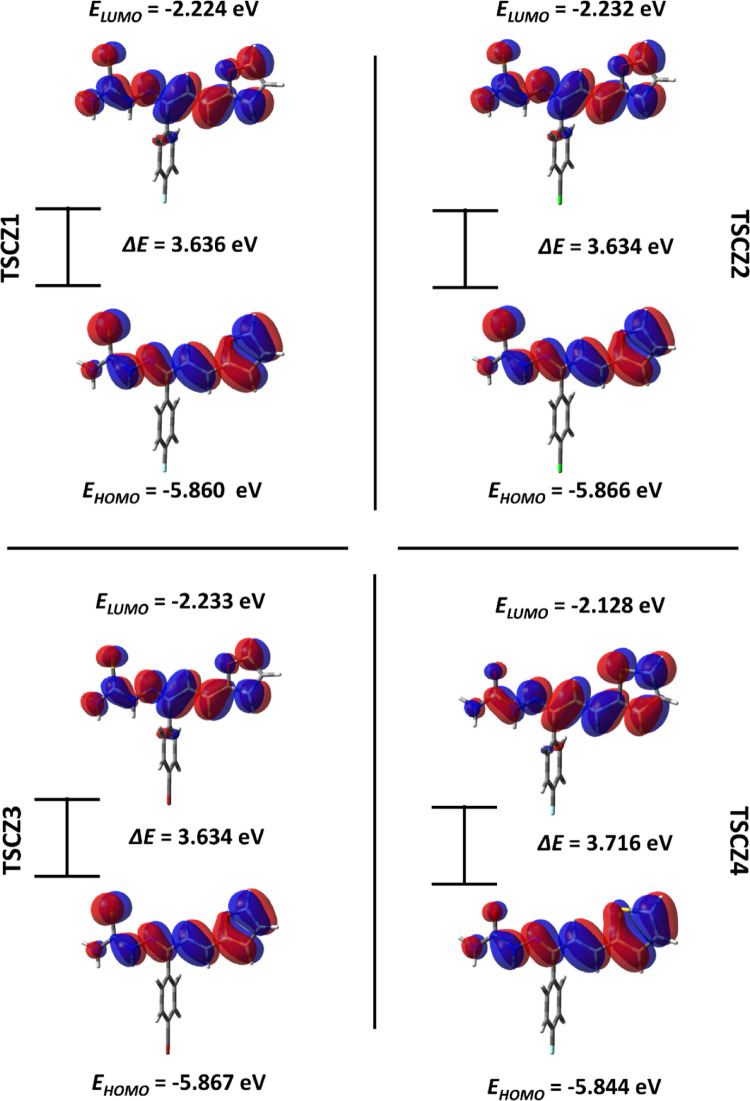
Frontier Molecular Orbital for TSCZ derivatives calculated in Water

The FMOs for the TSCZ derivatives are shown in Fig. 2. For the TSCZ derivatives, the HOMO orbitals are primarily distributed in the π bonds of the thiosemicarbazone or semicarbazone groups, the unsaturated linear chain, and the furan or thiophene groups, characterizing them as the leading sites for electron density donation. On the other hand, the LUMO orbitals are distributed in the π* bonds of the same groups. The π bonds of benzene do not contribute to the formation of FMOs due to the orthogonality of benzene, causing the π bonds to be out of plane.

Energy analysis of the FMOs shows that derivatives 1–3 have a lower donor character and a higher electron density acceptor character than derivatives 4–5. The presence of F atoms increases the donor character and decreases the acceptor character; in contrast, the presence of Br atoms decreases the donor character and increases the electron density acceptor character. The energy values of the HOMO orbitals follow the trend: E_TSCZ3_ (-5.867 eV) < E_TSCZ2_ (-5.866 eV) < E_TSCZ1_ (-5.860 eV) < E_TSCZ6_ (-5.851 eV) < E_TSCZ5_ (-5.850 eV) < E_TSCZ4_ (-5.844 eV). The energy values of the LUMO orbitals follow the trend: E_TSCZ3_ (-2.233 eV) < E_TSCZ2_ (-2.232 eV) < E_TSCZ1_ (-2.224 eV) < E_TSCZ6_ (-2.137 eV) < E_TSCZ5_ (-2.136 eV) < E_TSCZ4_ (-2.128 eV).

Although the F atom is the most electronegative, it donates electronic density to the ring by mesomerism due to the similarity of the size of the orbitals with carbon, attenuating the withdrawing character. Therefore, derivatives containing F have higher E_HOMO_ and E_LUMO_ values in their respective groups. Conversely, the Br atom has a greater withdrawing character because it does not donate electron density to the ring due to the lack of similarity in the size of the orbitals with the carbon. Hence, derivatives containing Br have lower E_HOMO_ and E_LUMO_ values in their respective groups.

In addition, the E_HOMO_ and E_LUMO_ values are used to calculate the energy gap (Δ*E*), Eq. 1, in which this parameter is used as a measure of chemical stability for the analysis of electronic transitions and characterization of structures such as hardness and softness (Arjunan et al. 2011). The results obtained indicate that the energy gap values increase in the sequence: TSCZ3 = TSCZ 2 < TSCZ1 < TSCZ6 = TSCZ5 < TSCZ4; this order suggests that the derivatives containing the semicarbazone group are more stable and less polarizable, this observation can be justified by observing the sulfur and oxygen atoms of which the sp^2^ sulfur atom is more polarizable in the thiosemicarbazones than the sp^2^ oxygen in the semicarbazones, reflecting in lower energy gap values.

#### Global chemical reactivity descriptors (GCRDs)

To understand the overall chemical behavior, chemical reactivity descriptors were calculated from the HOMO and LUMO energy values. Table S2 shows the calculated descriptor values for the TSCZ derivatives. The ionization potential measures the resistance of a molecule to losing an electron. Conversely, electronic affinity measures the ability of a molecule to gain an electron (Kumer et al. 2017); simultaneously, χ is associated with charge transfer between molecules, constantly occurring from the region of lowest electronegativity to the region of highest electronegativity (Almeida-Neto et al. 2020).

Thus, the increase in *I*, *EA*, and *χ* values follows the same sequence: TSCZ4 < TSCZ5 < TSCZ6 < TSCZ1 < TSCZ2 < TSCZ3. Although the structural changes have little effect on the *I*, *EA*, and *χ* values, the semicarbazone groups’ derivatives have a greater electron-donating character and a lower electron-accepting character. Regarding substitutions with halogens, derivatives containing F show greater donor character, while derivatives containing Br show greater resistance to electron donation. Conversely, derivatives containing thiosemicarbazone groups have a higher electron-accepting character, and substitution with Br increases this character.

Overall hardness (η) and overall softness (*S*) are parameters based on Pearson’s Maximum Hardness Principle (MHP), which states that molecules organize themselves electronically in such a way as to maximize hardness (Miar et al. 2021). These descriptors are related to the energy gap, in which higher energy gap values indicate higher hardness values and lower softness values (Noorizadeh 2005). The increase in η follows the order: TSCZ3 = TSCZ2 < TSCZ1 < TSCZ6 = TSCZ5 < TSCZ4, corroborating the data obtained from the energy gap.

In each group (thiosemicarbazones and semicarbazones), the derivatives show the same values for S (TSCZ1 = TSCZ2 = TSCZ3 = 0.550 eV; TSCZ4 = TSCZ5 = TSCZ6 = 0.538 eV), indicating an inverse trend and greater softness for the thiosemicarbazone derivatives. Substitution with halogens (F, Cl, and Br) did not significantly affect the η and *S* values.

The indices of nucleophilicity (ϵ) and electrophilicity (ω) were calculated to measure the nucleophilic and electrophilic character, respectively. The results show that the ω values obtained follow the trend: TSCZ4 < TSCZ5 < TSCZ6 < TSCZ1 < TSCZ2 < TSCZ3. All the derivatives have a higher electrophilic character, as the ω values are higher than ϵ. Derivatives containing the thiosemicarbazone group have greater electrophilic character and the presence of substitution with Br increases this character, corroborating the results obtained for *EA* and χ. Substitution with F increases the nucleophilicity of these derivatives, corroborating the results obtained for I.

#### Fukui’s condensed fuction

The chemical behavior of each atom can be analyzed through the Condensed Fukui Functions $\:{f}_{A}^{+}$ e $\:{f}_{A}^{-}$and using the Hirshfeld atomic charge population analysis given by Eqs. 9 and 10, respectively. Tables S3 and S4 show the Fukui functions for the TSCZ derivatives. These parameters are used to calculate the dual ($\:\varDelta\:f$)and multiphilic (Δ*w*) descriptors, which simultaneously and unequivocally discriminate the chemical behavior of each atom.

Tables S5 and S6 show the calculated values of and Δw for the TSCZ derivatives. From this data, Fig. S2 was constructed, showing the variation in the values of the multiphilic descriptor for each structure. The results indicate that the TSCZ1 and TSCZ2 derivatives show identical chemical behavior profiles, in which the N3, C2, C3, C4, C2’, C3’, C4’, C5’, C6’, F or Cl, C’’, C2’’, C3’’ and C4’’ atoms exhibit greater electrophilic character, and the C, N1, N2, C1’ and S atoms show greater nucleophilic character.

The TSCZ3 derivative shows a different profile, with the C1’, C2’, C3’, C4’, C5’, C6’ and Br atoms showing electrophilic character, and the C1, C2, C3, C4, N1, N2, N3, C1’’, C2’’, C3’’, C4’’, O and S atoms showing nucleophilic character. The derivatives with the semicarbazone group show identical chemical behavior profiles, in which the C1, C2, C4, N3, C1’, C2’, C3’, C4’, C5’, C6’, F, Cl, Br, C2’’ and S atoms show electrophilic character, and the C3, N1, N2, O, C1’’, C3’’ and C4’’ atoms show nucleophilic character.

#### Molecular electrostatic potential (MEP)

Analysis of the Molecular Electrostatic Potential (MEP) surfaces indicates the distribution of electronic density in the target molecule by color, where warmer colors such as yellow, orange, and red denote greater electronic density in the sequence from left to right, while cooler colors such as green and blue denote lower electronic density in the sequence from left to right (Da Silva et al. 2024). This distribution of electronic density represents the separation of electric dipoles in covalent bonds, which can be influenced by the electronegativity and size of the atoms, as well as the analysis environment, where it can be observed that more polar environments tend to increase the separation of electric dipoles.

**Fig. 3:**
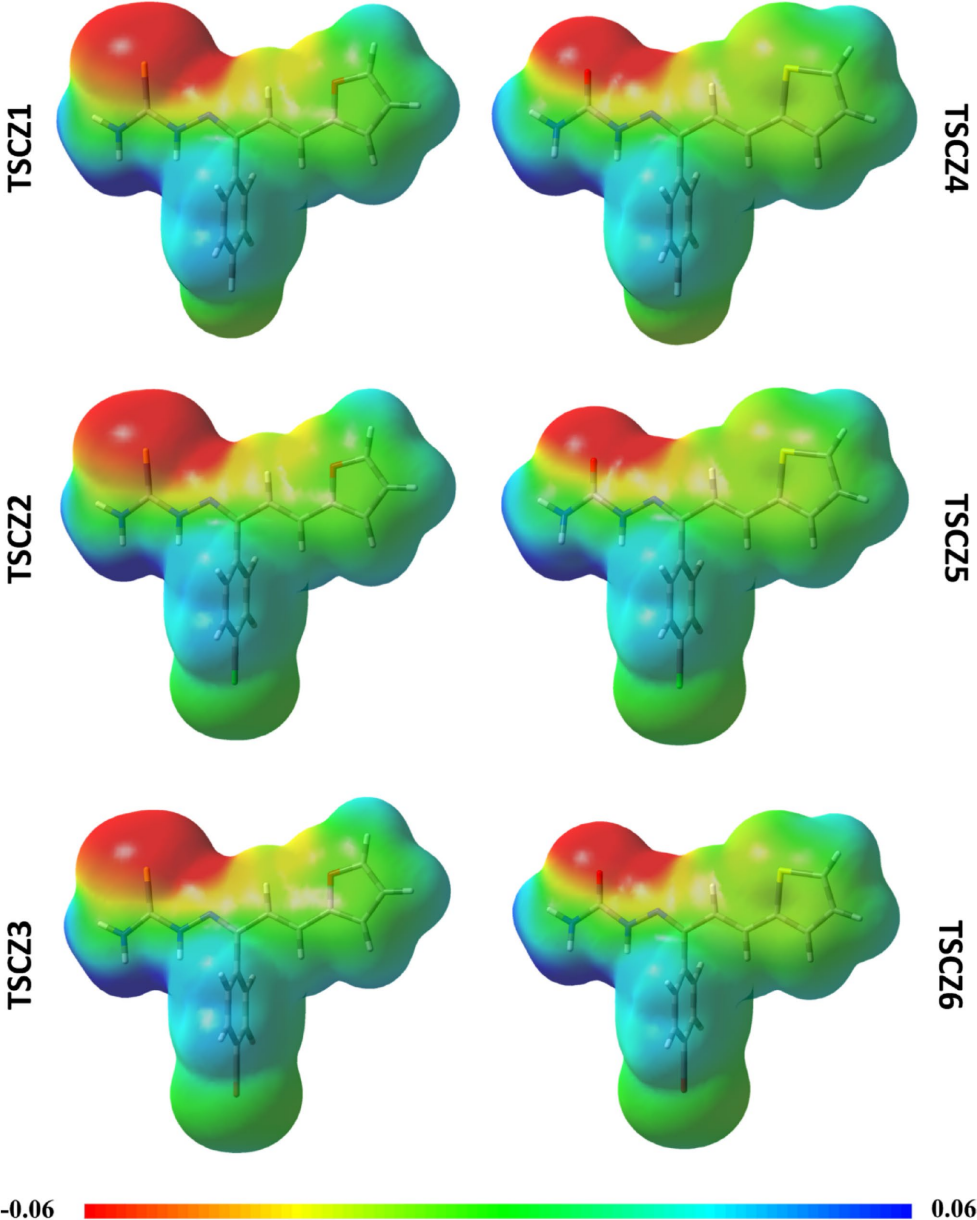
Potential eletrostatic molecular surface (MEP) for TSCZ derivatives in water

The MEP for the TSCZ derivatives was calculated in water to simulate the biological analysis environment (Fig. 3). The regions with the lowest electronic density are located on the hydrogens attached to the thiosemicarbazone group (TSCZ1-3), semicarbazones (TSCZ4-6), and the hydrogens of the benzene ring of all the derivatives. In addition, the regions with the highest electron density are located on the sulfur and nitrogen atoms of the thiosemicarbazone derivatives and the oxygen and nitrogen atoms of the semicarbazone derivatives.

The presence of the polar thiosemicarbazone and semicarbazone groups induces the formation of a dipolar moment in their direction, resulting in a higher electronic density on the sulfur atom for the TSCZ1-3 derivatives and on the oxygen atom for the TSCZ4-6 derivatives. The high electronic density of the sulfur atom in the TSCZ1-3 derivatives can also be explained by the fact that it is sp^2^ hybridized and has a larger atomic radius and polarizability, while the non-bonding electrons of the oxygen in the furan group participate in the resonance of the ring, causing a decrease in electronic density on this atom. The opposite can be observed for the semicarbazone derivatives, in which the oxygen atom shows greater electron density.In this case, the oxygen atom has sp^2^ hybridization, and the sulfur atom in the thiophene ring participates in the resonance, resulting in a lower electronic density. The substituents on the benzene ring moderately affect the electronic density of the aromatic hydrogens, where it can be seen that the presence of the most electronegative substituent (F) induces the formation of a surface with a lower electronic density on the aromatic hydrogens, while the presence of the least electronegative substituent (Br) induces the formation of a surface with a higher electronic density.

### (Eco)Toxicological analysis

Considering the impacts of thiosemicarbazones application as insecticides on the central nervous system, Table S7 was drawn up using data on the compounds physicochemical, environmental, and ecotoxicological properties, as well as their acute/chronic toxicity in aquatic organisms.

Table S7 shows that the log_Kow_ of the thiosemicarbazones varies between 4.2 and 5.385, indicating that these molecules are lipophilic (log_Kow_ > 3), i.e., they tend to have low solubility and adsorb to organic matter and sediment (Wong et al. 2021).

In agreement, the insecticides TSCZ1 and TSCZ4-6 showed high persistence in sediment, with a half-life of 227 days, TSCZ3 for 127 days, and TSCZ2 showed no persistence, lasting up to 72 days. This persistence may also be associated with the furan and thiophene fragments in thiosemicarbazones, contributing to a longer environmental half-life (Hijazi and Bottaro 2018; Langa; Kibet and Francis, 2024). In the other compartments, the compounds did not show persistence, with half-lives ranging from 34 to 74 days in soil, while in water, they remained between 5 and 22 days, Table S7.

Exposure to these lipophilic insecticides, even for a few days, can result in their accumulation in aquatic organisms, as substances with a log_Kow_ greater than three are potentially bioaccumulative due to their affinity with lipids (Gimeno et al. 2024). Bioaccumulation potential can be determined in fish employing BCF and BAF, where the former refers to the concentration of the toxicant absorbed by the respiratory and dermal routes; at the same time, the latter is related to absorption by all routes of exposure, including food (Del Carmen Gómez-Regalado et al. 2023).

The values show that thiosemicarbazones have a BCF of between 83.1 and 796 L/Kg, as well as a BAF of between 83.11 and 820.3 L/Kg, which are considered to be below the ideal (BCF/BAF ≥ 5000 L/Kg) for bioaccumulative molecules (Arnot and Gobas 2003). However, the bioconcentration process eventually occurs due to BCF, BAF, and logKow. Potentially bioconcentrated substances, such as TSCZ1-3, showed a half-life in fish between 1.325 and 2.082 days, while TSCZ4-6 showed a half-life between 0.204 and 0.321 days, Table S7. This short period is also possibly associated with the low persistence of thiosemicarbazones in water, where there is little interaction with these living beings.

As a result of exposure to these insecticides, the acute toxicity in fish and *D. magna* is high primarily since, in the former, the concentrations of TSCZ1-3 and TSCZ6 vary between 0.263 and 0.903 mg/L. In crustaceans, this toxic effect is denoted by TSCZ1-3, TSCZ5, and TSCZ6 with LC50s of 0.204 to 0.768 mg/L.

The other molecules showed moderate toxicity in fish, with concentrations equivalent to 2.511 (TSCZ4) and 1.059 mg/L (TSCZ5), and in *D. magna*, the LC50 of TSCZ4 corresponds to 1.747 mg/L. In *G. algae*, only TSCZ2 and TSCZ3 showed a high inhibition effect with EC50 equal to 0.713 and 0.553 mg/L, while the other substances showed results with a moderate inhibitory effect between 1.216 and 3.014 mg/L, Table S7.

For chronic exposure in fish, the thiosemicarbazones TSCZ1, TSCZ4, and TSCZ5 showed moderate toxicity with ChV between 0.118 and 0.312 mg/L, and TSCZ2, TSCZ3, and TSCZ6 showed high toxicity with concentrations ranging from 0.037 to 0.098 mg/L. In *D. magna*, only the molecules TSCZ2 and TSCZ3 showed high toxicity with ChV of 0.063 and 0.047 mg/L. The remaining compounds (TSCZ1 and TSCZ4-6) showed a moderate toxic effect with values between 0.111 and 0.3 mg/L. Finally, in *G. algae*, all the insecticides showed moderate biomass inhibition, corresponding to a concentration range between 0.290 and 1.241 mg/L, Table S7.

The concentrations of certain thiosemicarbazones were higher than their solubility, which results in different consequences depending on the organism. For example, TSCZ2, TSCZ3, TSCZ5, and TSCZ6 can generate a minimal inhibition effect on the growth of *G. algae*, but the potential effect on fish and *D. magna* is the narcosis caused by TSCZ3 and TSCZ6.

However, as the logKow value (5.385) of TSCZ3 exceeds the limits established by ECOSAR^®^ (5.0 for fish and *D. magna*), the narcosis is possibly reversible as it is not perpetuated in the chronic toxicity test, as a consequence of the log_Kow_ exceeding the determined limit resulting in no effect on the saturation of the compound (Tolls et al. 2009).

Chemicals that induce narcosis are characterized by their acute elemental toxicity, which results from their accumulation in living beings, suspending molecular interactions in cell membranes, leading to decreased respiration, lethargy, impaired balance, and later death (Matsa et al. 2019; Massei et al. 2021). Structurally, the fragments that cause the narcotic effect of thiosemicarbazones are ethers, alkyl halides, substituted benzenes, and possibly thiophene and furan cycles, which are essential structures for the conformation of the molecules in question (Adhikari and Mishra 2018; Veith et al. 1983).

### Screening of target binding and CNS MPO

The implemented screening model integrates 3D/2D analyses with previously characterized ligands to estimate the biological activity and interaction of small molecules and secondary metabolites with enzymes in the physiological context of humans or other phylogenetic lineages, Table S8.

**Fig. 4:**
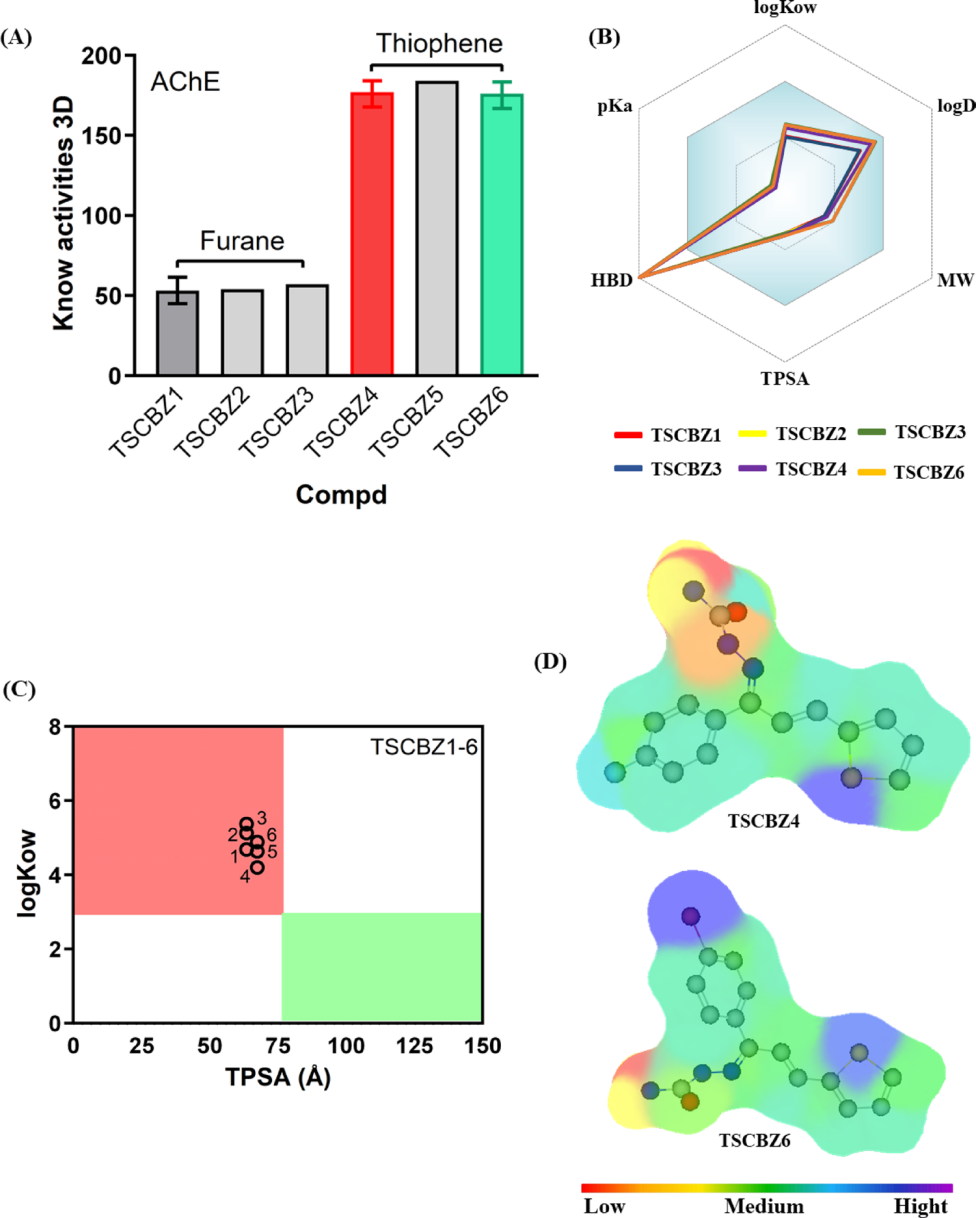
**A** 3D similarity test of TSCBZ1-6 via AChE, **B** CNS MPO scores of TSCBZ1-6, **C** Relationship between TPSA and log_Kow_ to estimate CNS access and **D** molecular lipphilicity potential (gradient of variation of low molar surfaces, red to yellow color) and high molar surfaces (green to blue color)

These results indicate that TSCBZ1-6 are promising candidates for hydrolase ligands, which correspond to approximately 50% of the bioactivity of these compounds with these target classes expressed in the *Mus musculus* system. This modality is associated with the structural similarity between TSCBZ and data available in ChEMBL, Table S8.

Among the studied compounds, TSCBZ4 and TSCBZ6 exhibited the highest similarity to known AChE ligands using the ligand-based method. Specifically, 176–177 structurally related compounds were identified, characterized by halo-substitutions (-Cl, -F, -Br) and containing thiazole or triazole substructures, as well as sulfonamide or thiophene groups (Fig. 4A).

In general, TSCBZ1-6 belonged to the class of compounds with a CNS MPO score ranging from 4.05 ~ 4.71, associated with the balance between physicochemical descriptors, especially lipophilicity, and polarity, making up the ideal spectrum under the premise of brain permeation (Fig. 4B). In this way, the polar fragments of the thiophene and furan rings establish biosafety for the CNS, however, the presence of the HBD groups reduces this purpose, since they are potentially more polar groups plus the replacement of the F and Br atoms, increasing the polarity, consequently, enabling a cerebral permeability (Fig. 4D).

Therefore, the topological polarity (TPSA) and lipophilicity (adapted in log_Kow_) of TSCBZ1-6 corroborate the MPO score graph and DFT calculations, establishing that halo substitution (-Br, -Cl, and F) have a low significant effect but denote stability in which the semicarbazone and thiosemicarbazone skeletons provide greater relevance in brain permeability (Fig. 4C).

### Redocking between inhibitor GNT (galantamine) and ache

**Fig. 5:**
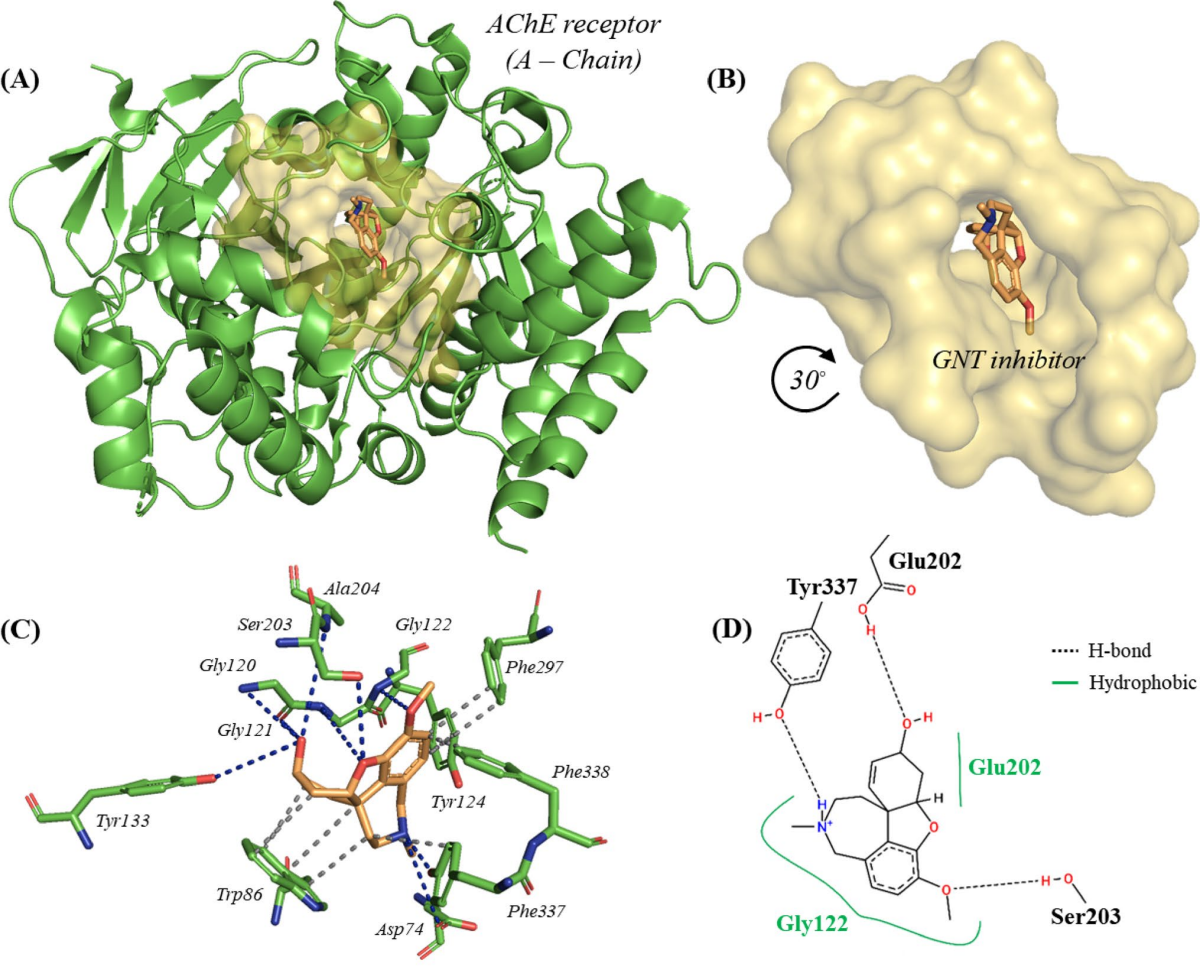
**A** Docking position of the GNT inhibitor at the AChE enzyme, **B** Cavity of the GNT inhibitor, **C** 3D map of the strongest interactions and **D** 2D map of the strongest interactions

Acetylcholinesterase (AChE) is a neurotransmitter that regulates neurotransmission in the synapses of the CNS (Central Nervous System) (Fig. 5A); however, inactivation of AChE causes lethal disturbances for any organism with a nervous system, so inhibitors applied to insecticides delimit great modulation in AChE/BChE (Silman 2021).

Cheung et al. (2012) reported that the inhibitor (-)-gantamine (GNT) creates a protein-ligand complex with the AChE enzyme. This interaction occurs with the residues Trp86, Tyr124, Phe297, Phe338, Asp74, Gly120, Gly121, Gly122, Tyr133, Ser203, and Ala204 with specificity for the Tyr337 residue that constitutes one of the catalytic sites for the inhibition or modulation of AChE, Fig. 5B and Table S9.

Thus, the inhibitor (-)-galantamine (GNT) interacted with four (4) hydrophobic residues Trp86, Tyr124, Phe297, Tyr337, and Phe338, of which the benzofuran portion contributed significantly to these interactions. Similarly, eight (8) hydrogen bonds (H-bonds) are described with residues Asp74, Gly120, Gly121, Gly122, Tyr337 (critical for AChE inhibition), Tyr133, Ser203, and Ala204 in which the pyridine substructure (amine portion) delimits significance (Fig. 5C), in addition, other 2D interactions were arranged between Gly122 (hydrophobic) and Glu202 (H-bond and hydrophobic), Fig. 5D and Table S9.

### Redocking between inhibitor 8U2 ((2-｛1-[4-(12-amino-3-chloro-6,7,10,11-tetrahydro-7,11-methanocycloocta[b]quinolin-9-yl)butyl]-1H-1,2,3-triazol-4-yl｝-N-[4-hydroxy-3-mehoxybenzyl]acetamide)) and BChE

Viayna et al. (2020) describes the cont h-potency entity in which the mechanism of BChE enables an adjacent view of the damage caused by insecticides on this enzyme, enunciating new (structure-based) approaches.

**Fig. 6:**
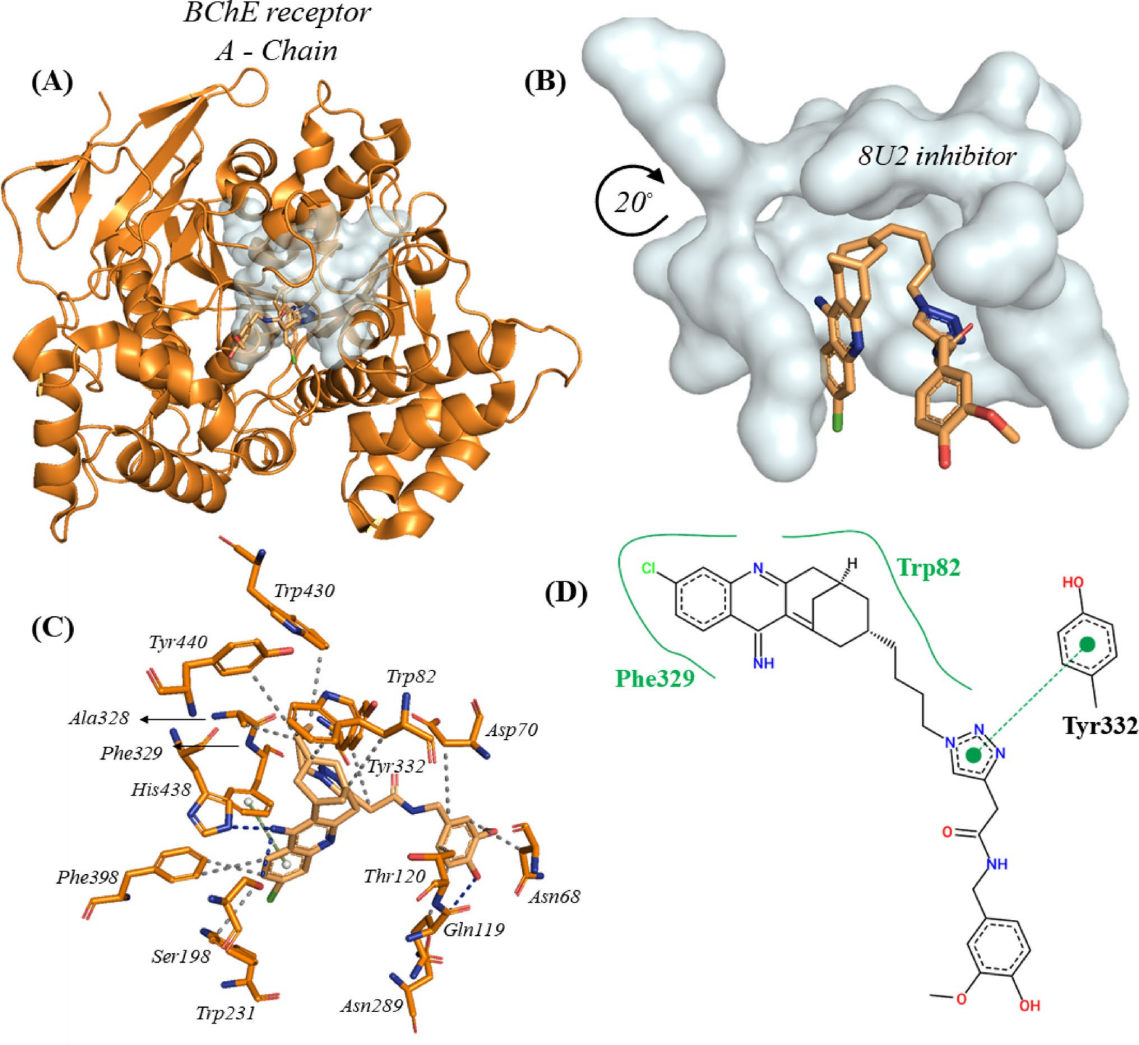
**A** Docking position of the 8U2 inhibitor at the BChE enzyme, **B** Cavity of the 8U2 inhibitor, **C** 3D map of the strongest interactions and **D** 2D map of the strongest interactions

Thus, Fig. 5A eludes a global perspective on the position of the inhibitor 8U2; however, Fig. 6B establishes that the catalytic site of BChE demonstrates a well-defined oval cavity comprising approximately ten (14) amino acid residues in its orthosteric site.

It is worth noting that the 8U2 inhibitor delimits twelve (12) hydrophobic interactions with the following residues: Asn68, Asp70, Trp82, Gln199, Thr120, Trp231, Ala328, Tyr332, Phe398, Trp430 and Tyr440, arranged mainly by the aliphatic chain and the quinolinimine group, which is added by the heteroatom (Cl). As well as three (3) hydrogen bonds with Ser198, Asn289 and His438 (Fig. 6C and Table S10), these established by the methoxyphenol portion and only one π-stacking interaction (parallel) identified by the triazole fragmente, Fig. 5D.

### Molecular dynamics studies ache

**Fig. 7:**
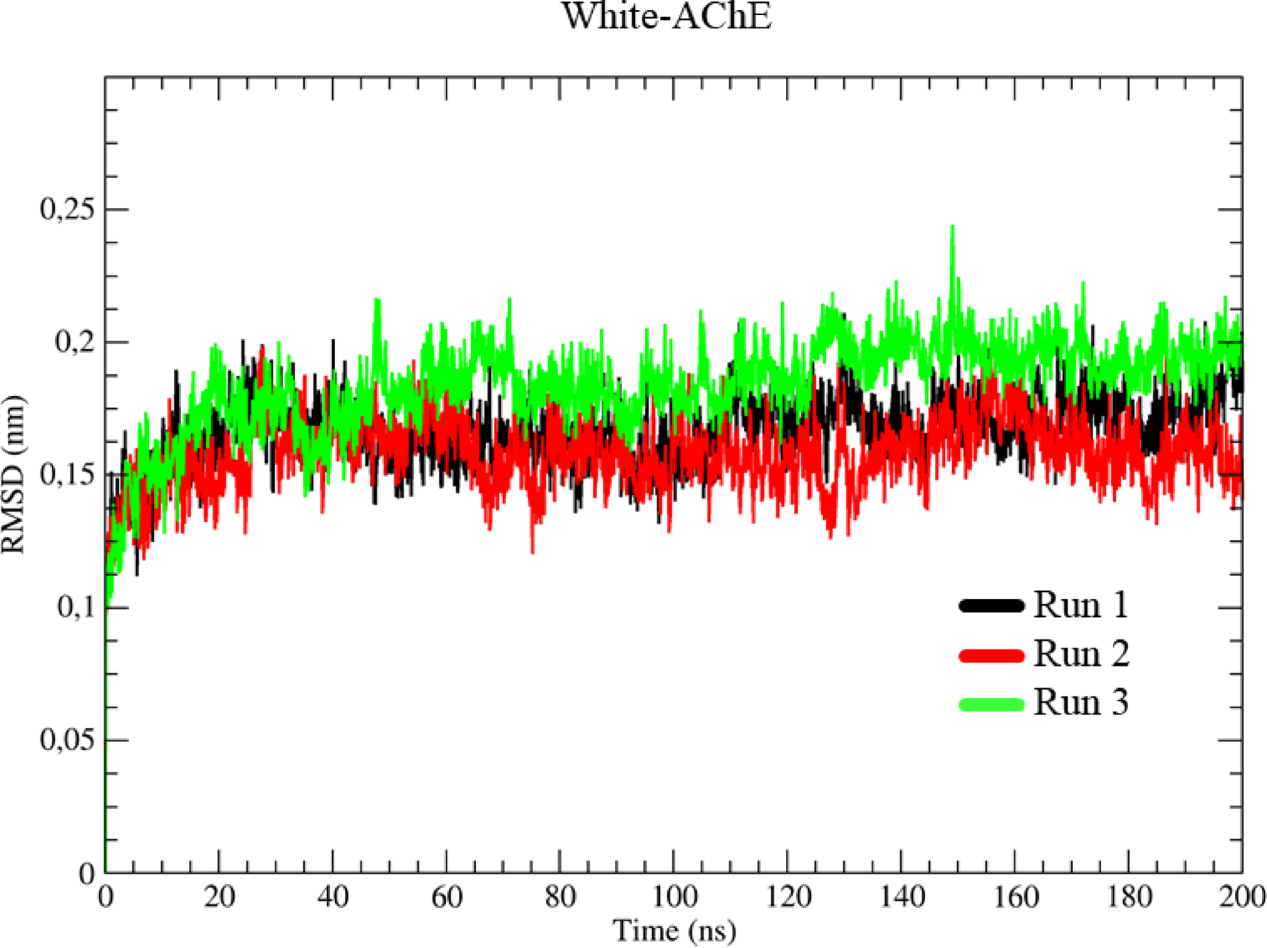
Determination of the root mean square deviation (RMSD) for the AChE, in three runs represented in black, red and green, respectively

The RMSD results for the clean protein showed that the three simulations (identified in black, red, and green, respectively, Run 1–3) showed similar behavior. At the start of the simulation, a variation of 1.40 Å was observed concerning the initial conformation (0 ns) until thermal equilibrium was reached at 1 ns, during which time the system’s temperature was increased and kept stable at 310 K, Fig. 7. After this phase, the three systems varied to 1.65 Å up to 20 ns, stabilizing at an average of 1.69 Å over 200 ns.

**Fig. 8:**
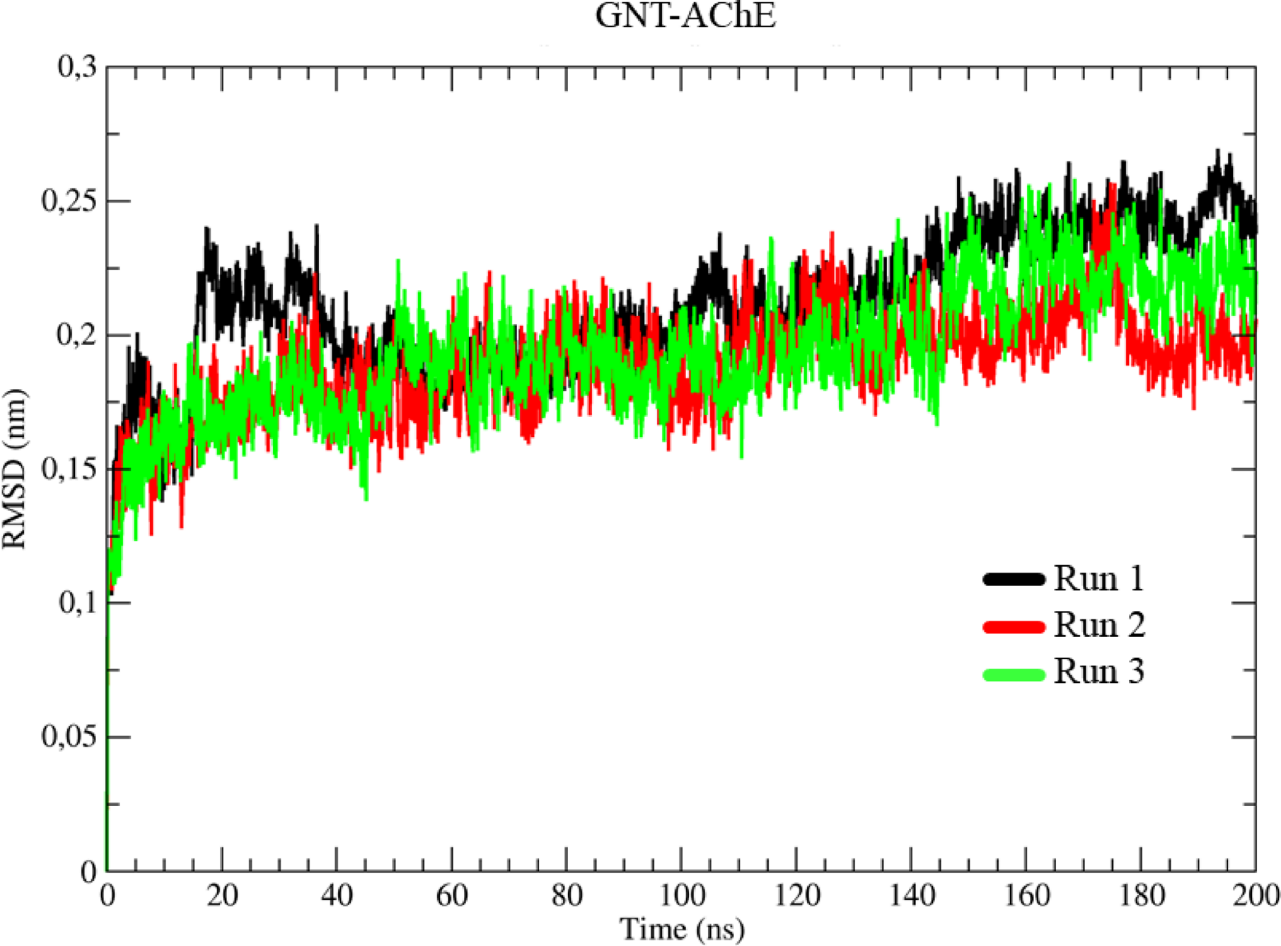
Determination of the root mean square of the deviation (RMSD) for the GNT-AChE complex, in three assays represented in black, red, and green, respectively

In order to gain a deeper understanding of the behavior of the system with the co-crystallized ligand, molecular dynamics (MD) simulations were carried out for the complex between the inhibitor and the acetyl protein (GNT-AChE), simulated in triplicates, represented in black, red and green (Run 1–3), Fig. 8. Analysis of the RMSD results for the GNT-AChE complex revealed an initial behavior similar to that observed for the AChE protein without ligand.

During the thermal equilibrium phase, the three simulations showed a variation of 1.50 Å. However, after this stage, the system with the inhibitor showed more significant conformational variation, especially between 20 and 45 ns, when a peak of 2.45 Å was recorded, followed by stabilization until 110 ns, when the variation remained relatively constant, with minor fluctuations. From 110 ns, the system began a gradual increase in variation, going from 1.65 Å to 2.0 Å between 110 and 140 ns and from 2.0 Å to 2.3 Å between 140 and 155 ns, stabilizing until the end of the simulation, Fig. 8.

**Fig. 9:**
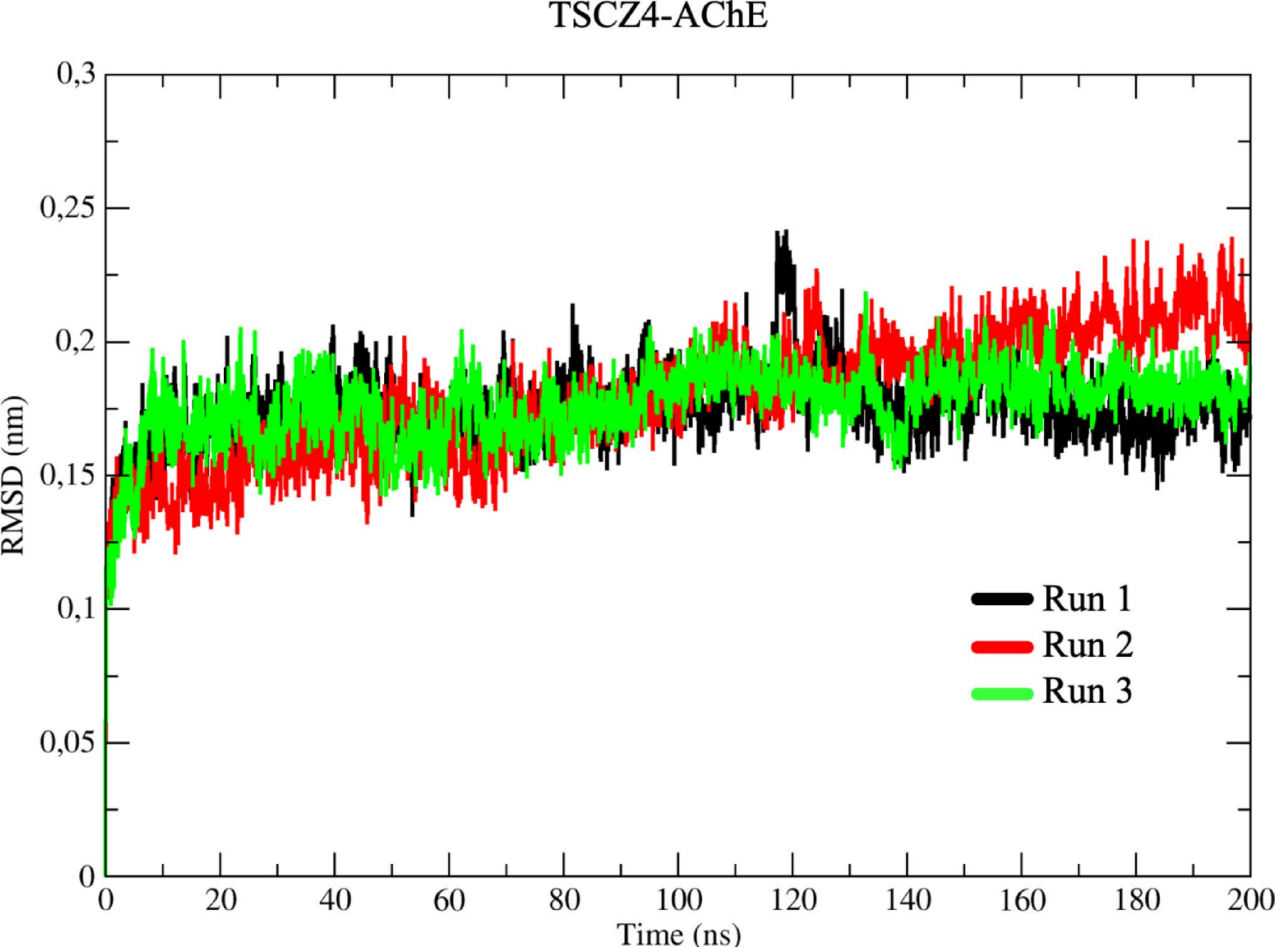
Determination of the root mean square of the deviation (RMSD) for the **TSCZ4-AChE** complex, in three assays represented in black, red, and green, respectively

As for the RMSD results for the system composed of the TSCZ4 ligand and the AChE protein (TSCZ4-AChE), represented in triplicates in the simulations (Run 1–3) in black, red, and green (Fig. 9), the initial variation during the thermal equilibrium phase was 1.25 Å in the first nanosecond. The system then increased by 0.05 Å, remaining stable until 70 ns. Between 70 ns and 120 ns, there was another slight increase of 0.05 Å in the conformation variation, and the system continued to show variations until the end of the simulation.

**Fig. 10:**
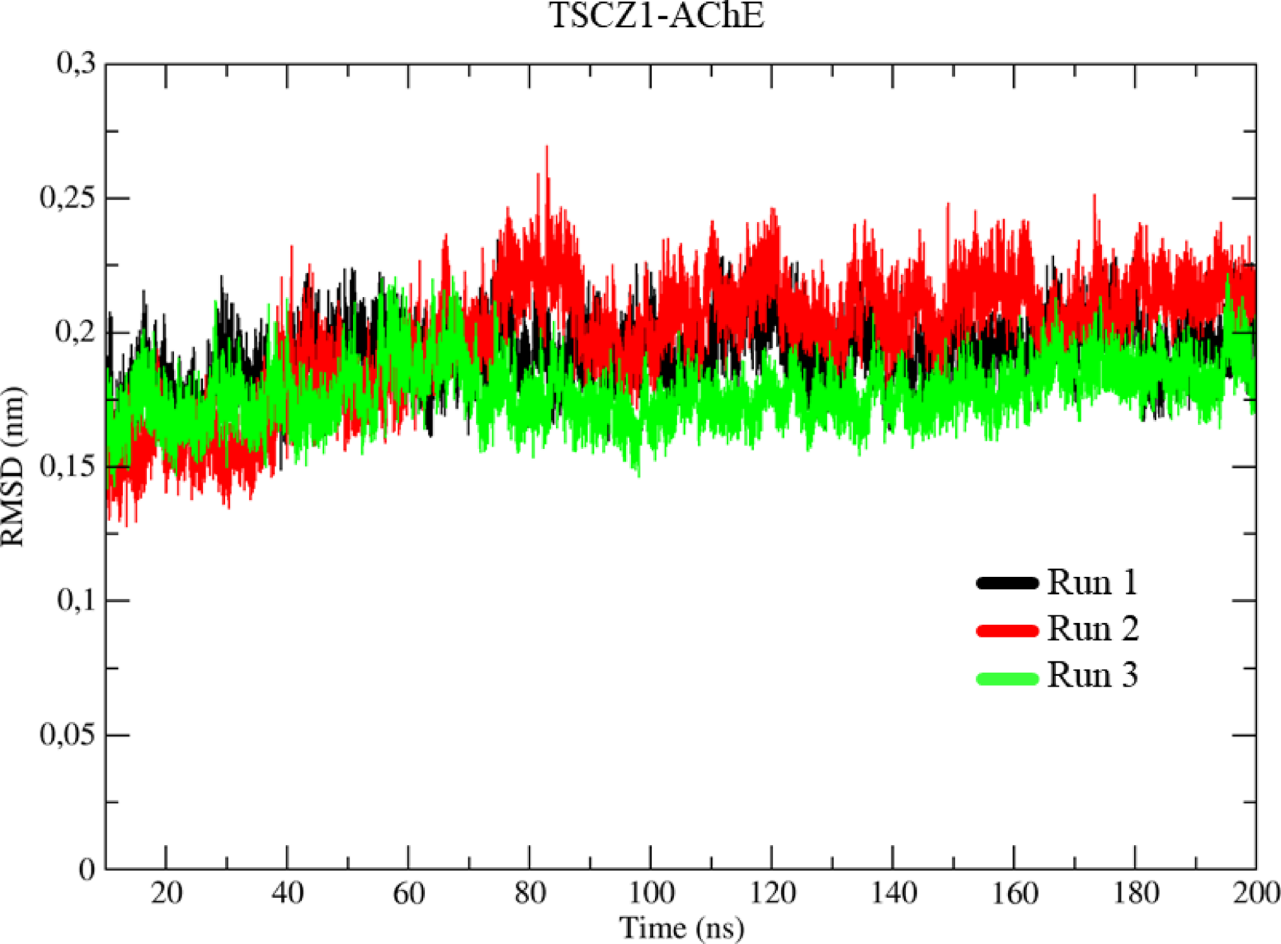
Determination of the root mean square of the deviation (RMSD) for the TSCZ1-AChE complex, in three assays represented in black, red, and green, respectively

As for the RMSD results for the system formed by the TSCZ1 ligand and the AChE protein (TSCZ1-AChE), represented in triplicates in the simulations (Run 1–3) in black, red, and green (Fig. 10), the initial conformation increases during the equilibrium stage, in a similar way to the other systems. The TSCZ1-AChE system shows a slight conformational variation after thermal equilibrium (1 ns) up to 20 ns. After this period, the system stabilizes, with an average of 1.75 Å, without showing significant conformational variations until the end of the simulation, at 200 ns.

**Fig. 11:**
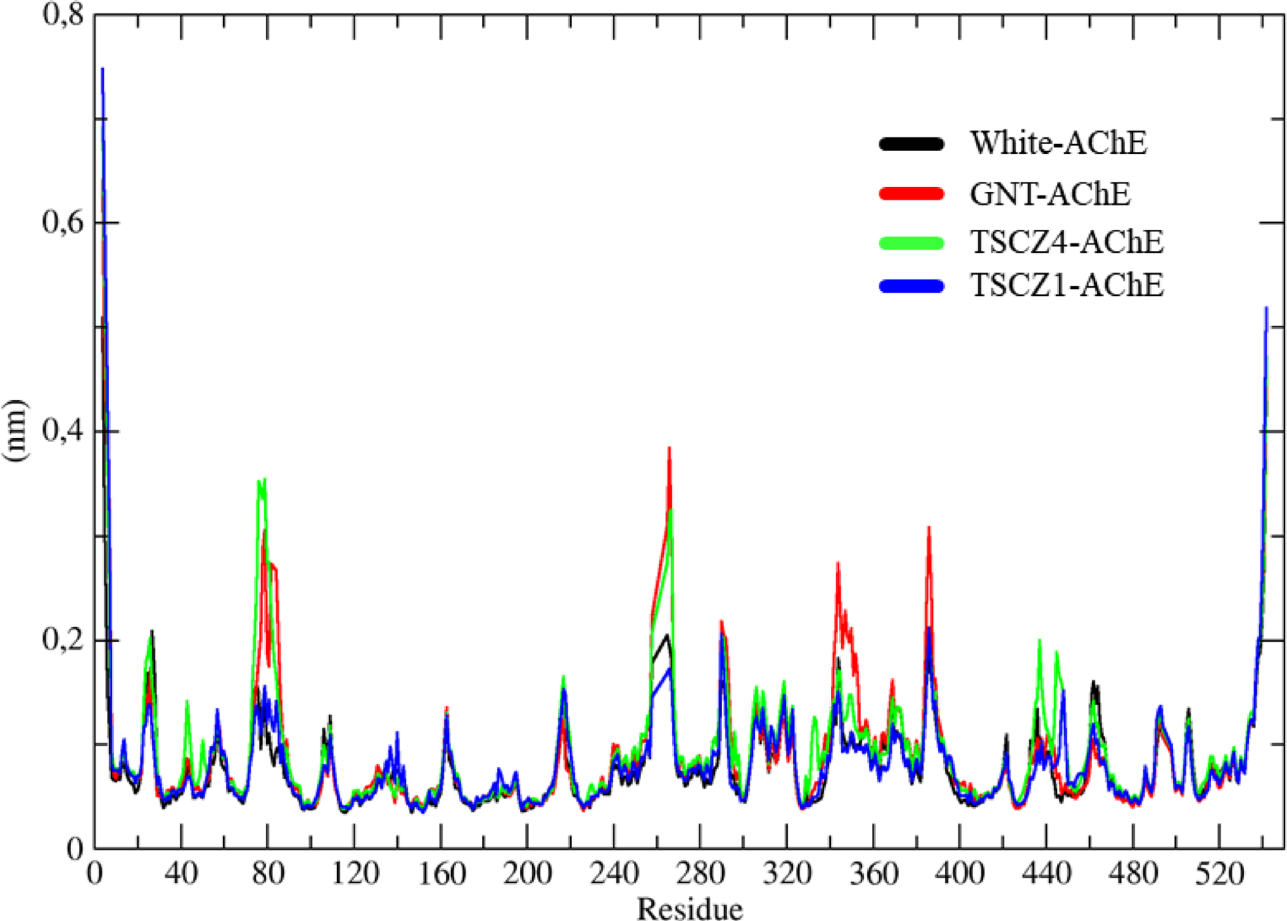
Determination of the root mean square fluctuation (RMSF) of the White-AChE, GNT-AChE, TSCZ4-AChE and TSCZ1-ZChE complexes represented in black, red, green and blue respectively

The RMSF results for the AChE systems, represented in black, red, green, and blue for the White-AChE, GNT-AChE, TSCZ4-AChE, and TSCZ1-AChE systems, respectively, show that the N-terminal Glu-3 residue varied by 7.5 Å, while the C-terminal residue varied by 5.2 Å (Fig. 11). Analyzing the RMSF values, it was observed that the White-AChE and TSCZ1-AChE systems (black and blue curves) showed the slightest variations, indicating a low conformational fluctuation.

In contrast, the GNT-AChE and TSCZ4-AChE systems (red and green curves) exhibited significant fluctuations in residues such as Phe-79, Val-269, Gly-335, His-386, Thr-435, and Ile-453, suggesting conformational changes in these specific residues. Thus, the TSCZ4 ligand showed conformational changes similar to those observed in the co-crystallized GNT ligand.

### MM/GBSA ache and occupancy of hydrogen bonds

The results of the energy components for ΔG_bind_ in the GNT-AChE, TSCZ4-AChE, and TSCZ1-AChE systems reveal significant differences in the interactions of each ligand with the target protein. The van der Waals energy term (E_vdW_) showed the following values: -41.56 for GNT-AChE, -43.94 for TSCZ4-AChE, and − 44.79 for TSCZ1-AChE.

The electrostatic energy (E_ele_) varied between − 5.33 and − 5.97, with TSCZ1-AChE showing the lowest value (-5.97), indicating an increase in electrostatic interactions for this system. The polar solvation energy (G_GB_) was slightly higher for TSCZ1-AChE (29.82) compared to the other systems, while the apolar solvation energy (GSA) ranged from − 3.11 to -4.01, with the most negative value observed for TSCZ4-AChE.

In addition, the entropic term (-TΔS) showed values of 4.83 for GNT-AChE, 6.32 for TSCZ4-AChE, and 5.83 for TSCZ1-AChE. In terms of ΔGbind binding energy, the values obtained were − 16.89 ± 2.16 for GNT-AChE, -17.81 ± 2.59 for TSCZ4-AChE and − 18.64 ± 2.32 for TSCZ1-AChE.

**Table 3:** Predicted binding free energy (kcal/mol) and individual energy terms of the GLN-AChE, TSCZ4-AChE and TSCZ1-AChE complexes

Term	GNT-AChE	TSCZ4-AChE	TSCZ1-AChE
E_vdW_	− 41.56	− 43.94	− 44.79
E_ele_	− 5,65	− 5,33	− 5,97
G_GB_	28.60	29.15	29.82
G_SA_	− 3.11	− 4.01	− 3.53
-TΔS	4.83	6.32	5.83
ΔG_bind_	−16.89 +/- 2.16	−17.81 +/- 2.59	−18.64 +/- 2.32

**Fig. 12:**
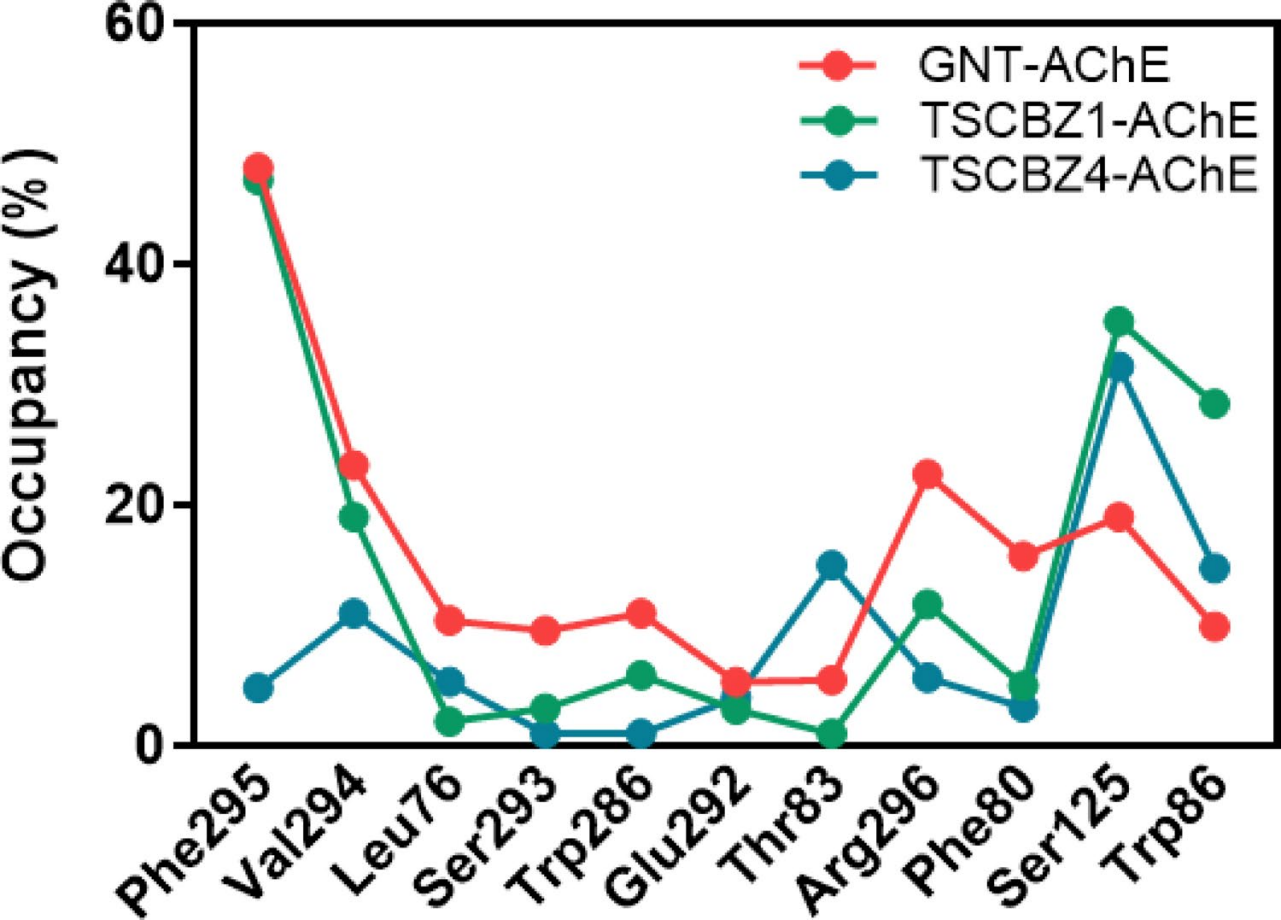
Hydrogen bond occupancy for GNT-AChE, TSCZ4-AChE and TSCZ1-AChE complexes in %

Analysis of the occupancy of the key residues in the GNT-AChE, TSCZ4-AChE, and TSCZ1-AChE systems revealed marked differences in the interaction profiles. The Phe-295 residue had an occupancy of 48% in GNT-AChE, 4.82% in TSCZ4-AChE, and 47% in TSCZ1-AChE. The Val-294 residue had occupancies of 23.34% in GNT-AChE, 11% in TSCZ4-AChE, and 19% in TSCZ1-AChE. However, Leu-76 showed reduced occupancy in all systems, with 10.4% for GNT-AChE, 5.33% for TSCZ4-AChE, and 2% for TSCZ1-AChE.

The residues Ser-293 and Glu-292 had occupancies of less than 10% in all systems. The residues Ser-125 and Trp-86 stood out in TSCZ4-AChE and TSCZ1-AChE, with occupancies of 31.52% and 14.79% for TSCZ4-AChE, and 35.29% and 28.43% for TSCZ1-AChE, respectively. In contrast, the Arg-296 residue had occupancies of 22.56% in GNT-AChE, 5.69% in TSCZ4-AChE, and 11.76% in TSCZ1-AChE, Fig. 12.

### Molecular dynamics studies BChE

**Fig. 13:**
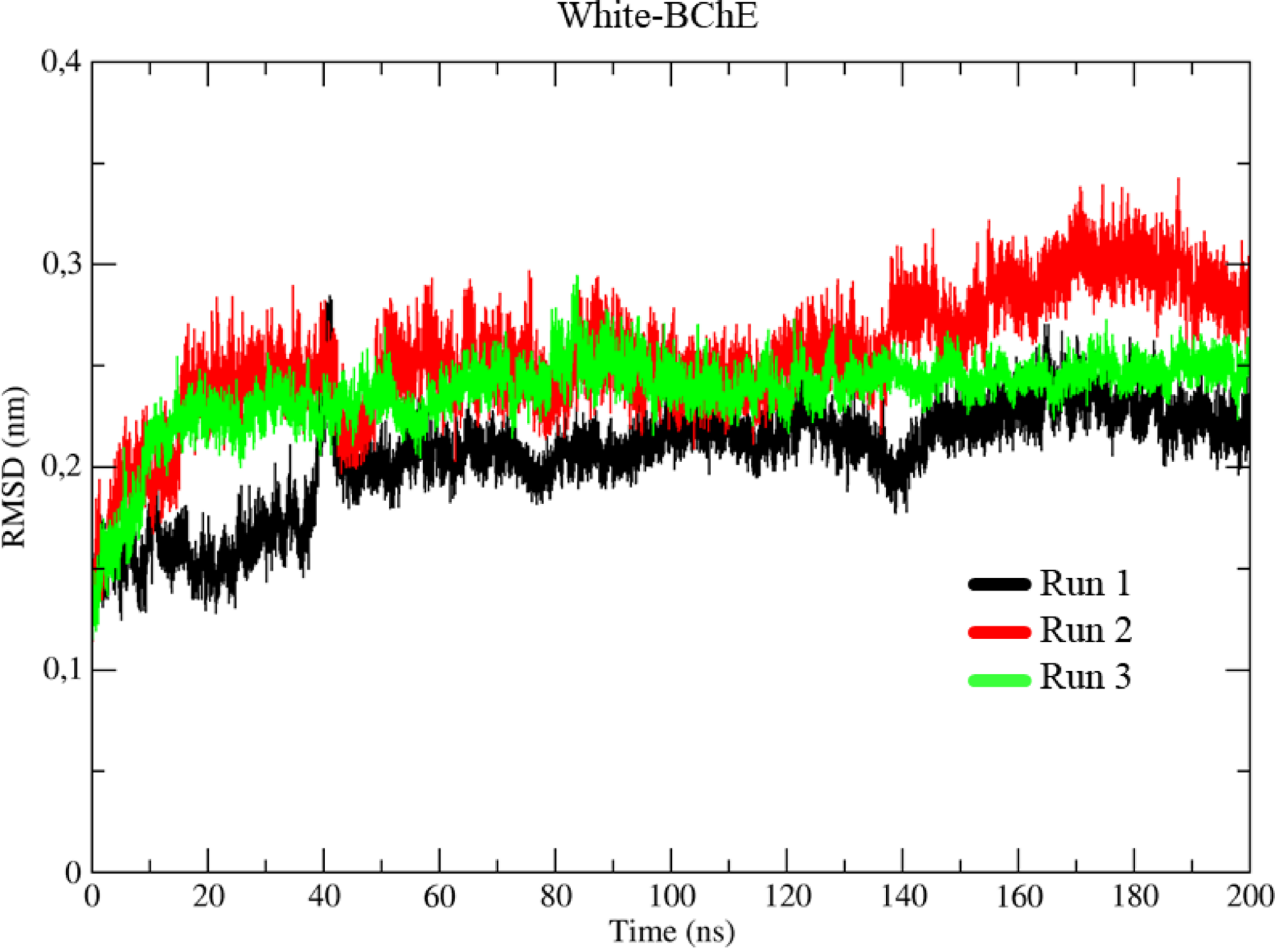
Determination of the root mean square deviation (RMSD) for the BChE, in three runs represented in black, red and green, respectively

Analysis of the trajectory files from the molecular dynamics (MD) simulations of the BChE protein made it possible to obtain the RMSD values over time to assess the protein’s conformational behavior without ligands or residues (Fig. 13). In the three simulations carried out (Run 1, Run 2, and Run 3), represented by the colors black, red, and green, a similar behavior was observed at the beginning, with a variation of 1.55 Å about the initial conformation (0 ns) until thermal equilibrium, reached around 1 ns, when the temperature was stabilized at 310 K.

After this equilibrium, the Run 2 and Run 3 simulations showed a variation of 2.30 Å between 1 and 40 ns, while Run 1 showed a minor variation of 1.57 Å. From 40 ns onwards, the three systems converged, with the conformational variations stabilizing at around 2.25 Å until approximately 140 ns. At this point, there was an increase in variation to 3.10 Å. After this increase, the conformational variation decreased again to around 2.20 Å, remaining stable until the end of the simulation, at 200 ns.

**Fig. 14:**
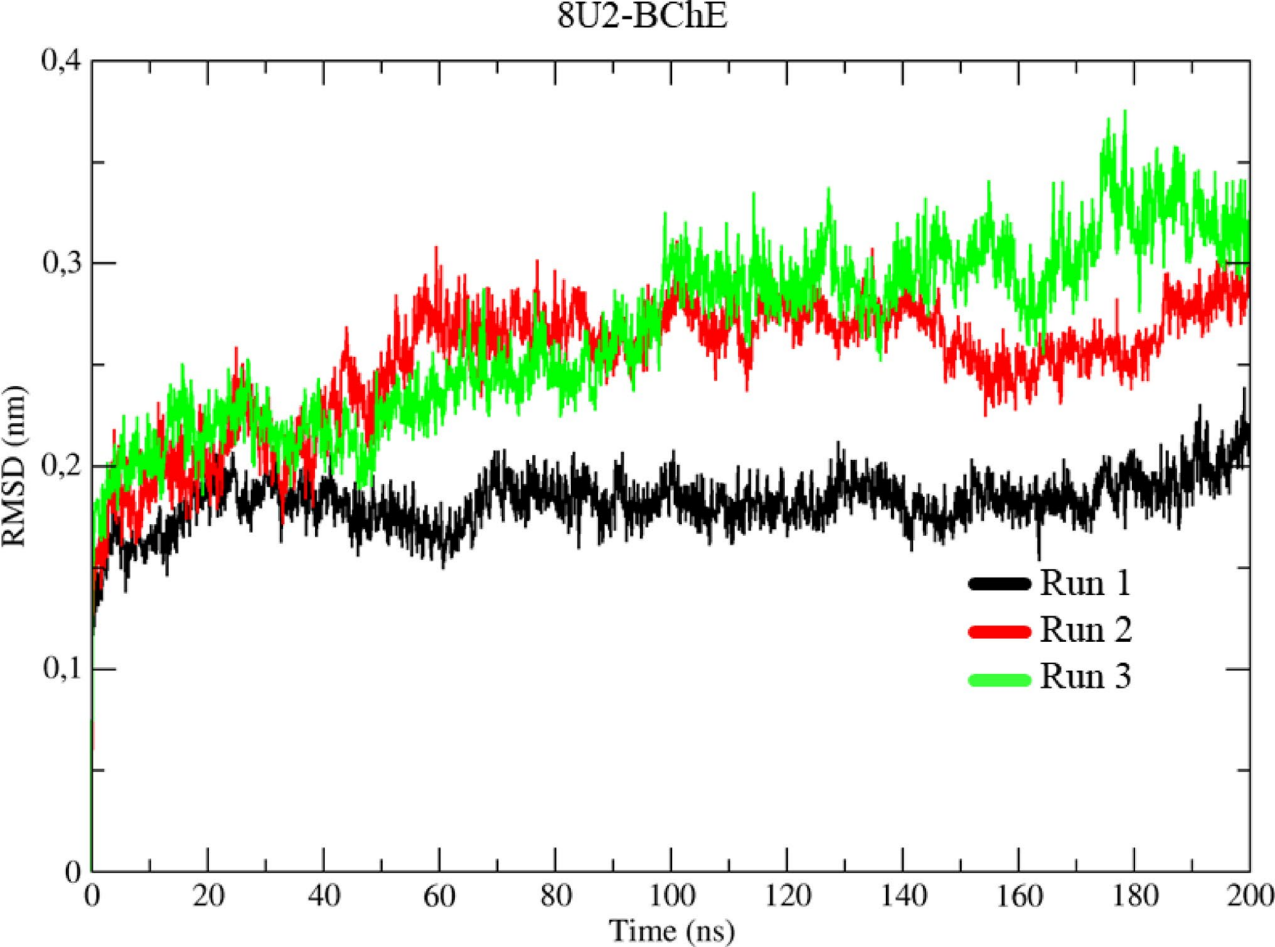
Determination of the root mean square of the deviation (RMSD) for the 8U2-BChE complex, in three assays represented in black, red, and green, respectively

The results of the molecular dynamics (MD) simulations of the BChE protein complexed with the 8U2 inhibitor, represented by the RMSD values in black, red, and green (Run 1–3) (Fig. 14), showed the following behavior. During the thermal equilibrium stage, a similarity was observed in the three simulations, with a variation of around 1.75 Å about the initial frame up to 1 ns. After this point, the system showed a conformational variation of around 2.0 Å, which lasted until approximately 40 ns.

From this time onwards, the Run 2 and Run 3 simulations showed an increase in conformational variation of around 0.75 Å, between 40 ns and 60 ns, followed by a phase of conformational stability between 60 ns and 85 ns. Between 85 ns and 95 ns, a new variation of 0.25 Å was observed. After this increase, the system maintained this stable conformation between 100 ns and 145 ns; at this point, a significant conformational change occurred, peaking at 3.75 Å. However, after this peak, the system returned to a conformational variation in the 2.5 Å range, remaining stable until the end of the simulation at 200 ns.

**Fig. 15:**
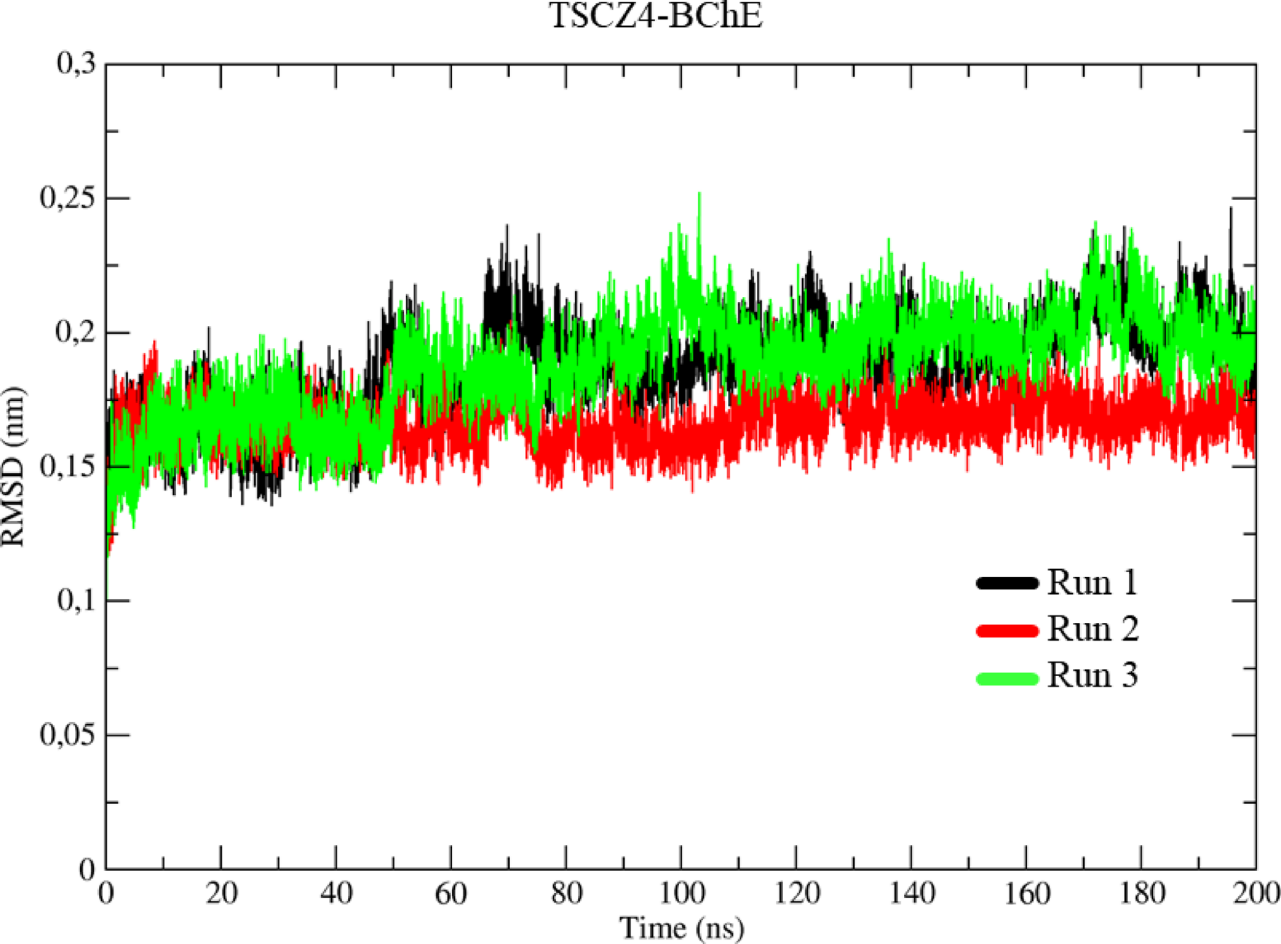
Determination of the root mean square of the deviation (RMSD) for the TSCZ4-BChE complex, in three assays represented in black, red, and green, respectively

The RMSD values obtained in the molecular dynamics (MD) simulations for the system with the TSCZ4-BChE complex, represented by the black, red, and green colors (Run 1–3) (Fig. 15), indicated the following behaviors. During the thermal equilibrium phase, a similarity was observed between the three simulations, showing an initial conformational variation of approximately 1.62 Å up to 1 ns.

After this point, the systems remained stable, with no significant conformational variations between 1 ns and 45 ns. Between 45 ns and 53 ns, there was a slight change of around 0.34 Å. After this peak, the conformational variations were minimal, with the three systems showing an average variation of 1.75 Å by the end of the simulation.

**Fig. 16:**
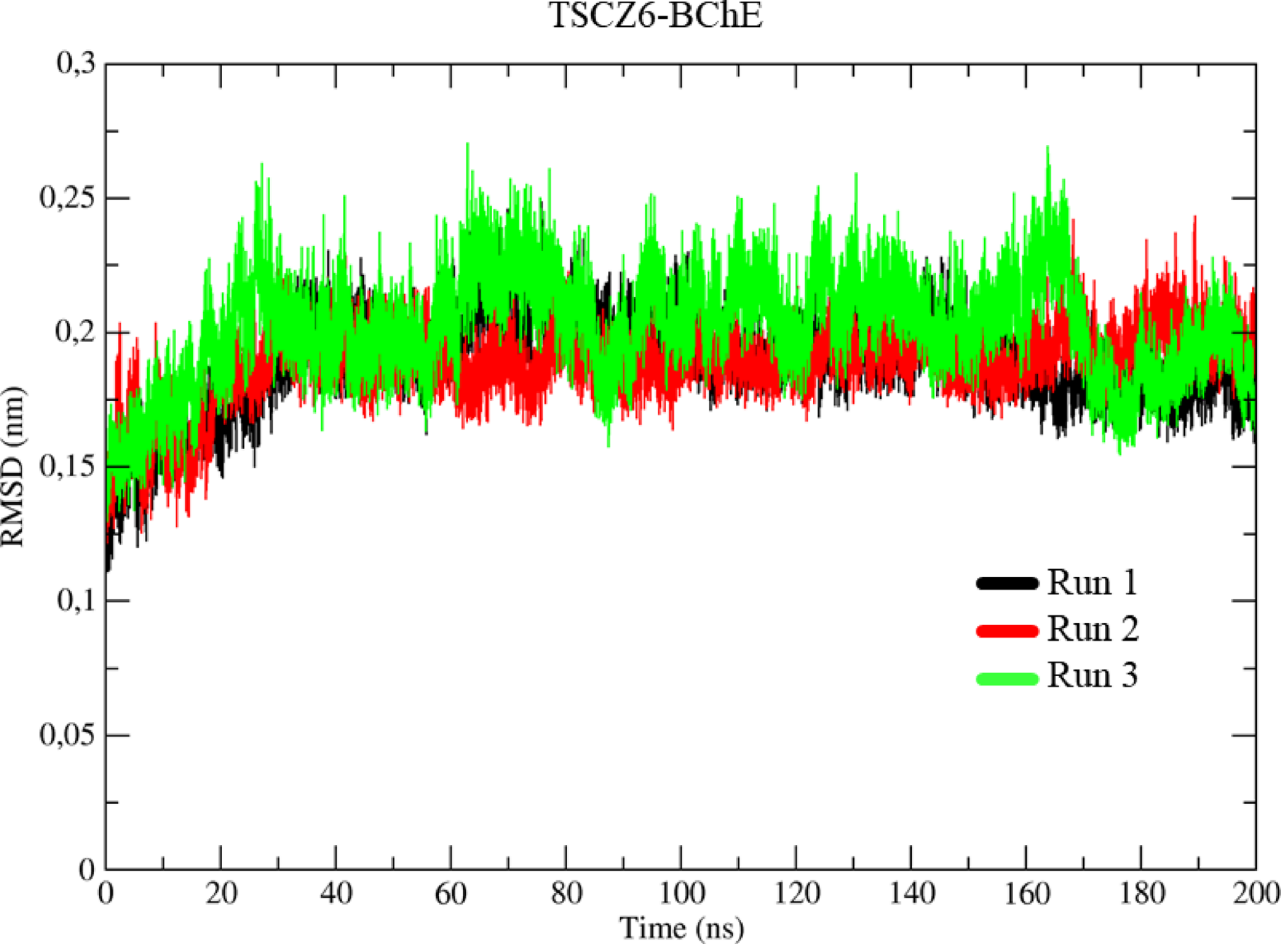
Determination of the root mean square of the deviation (RMSD) for the TSCZ6-BChE complex, in three assays represented in black, red, and green, respectively

The RMSD values for the TSCZ6-BChE complex in the molecular dynamics (MD) simulations are illustrated in Fig. 16, with the runs represented in black, red, and green (Run 1–3). It was observed that the behavior of the triplicates was similar. During thermal equilibrium, the initial variations were around 1.56 Å up to 1 ns, compared to the first frame (0 ns).

There was then an increase in conformational variation, reaching a peak of 2.58 Å between 1 ns and 30 ns. After this increase, the systems stabilized with an average of 2.05 Å until 160 ns, when a new change was recorded, with a variation of 1.75 Å. After this point, the conformational change was moderate, stabilizing at around 1.70 Å until the end of the simulation at 200 ns.

**Fig. 17:**
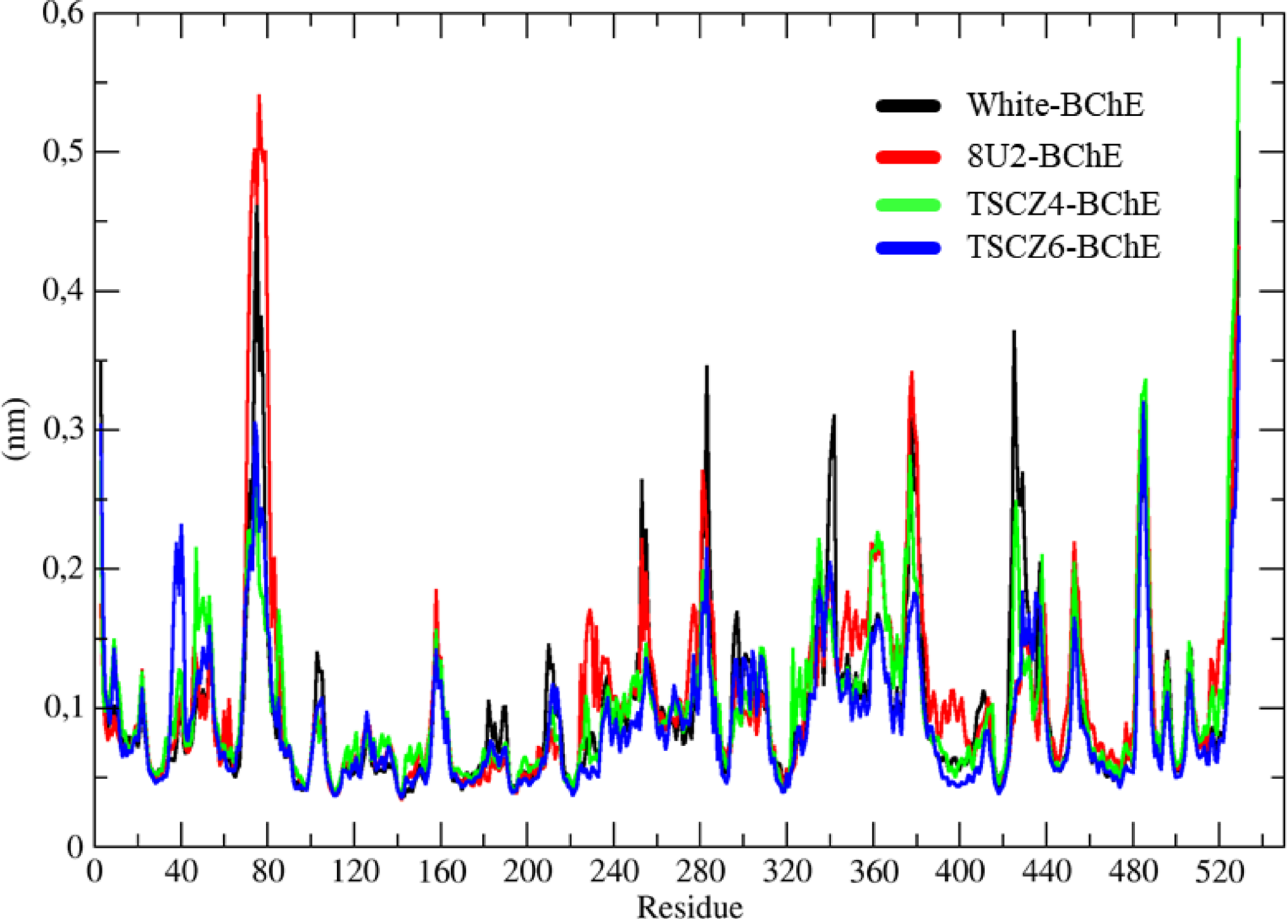
Determination of the root mean square fluctuation (RMSF) of the White-BChE, 8U2-BChE, TSCZ4-BChE and TSCZ6-BChE complexes represented in black, red, green and blue respectively

The RMSF (Root Mean Square Fluctuation) investigation evaluated the structural fluctuations of the BChE protein residues over time, reflecting the flexibility of the different regions. The White-BChE, 8U2-BChE, TSCZ4-BChE, and TSCZ6-BChE systems are represented in black, red, green, and blue, respectively (Fig. 17).

In all cases, more pronounced fluctuations were observed in the regions of the N-terminal residues, C-terminal residues, and surface loops. The Glu-108 residue, indicated on the graph as residue 80 [auth-80], showed a peak of variation in all the systems, particularly 8U2-BChE (red), which reached a maximum value of 5.74 Å.

The White-BChE and TSCZ6-BChE systems (in black and blue) showed more minor fluctuations, indicating more excellent conformational stability than the 8U2-BChE and TSCZ4-BChE systems. On the other hand, the 8U2-BChE and TSCZ4-BChE systems showed marked fluctuations in residues Ser-107 [auth 79], Pro-297 [auth 269], Gly-361 [auth 333], Arg-414 [auth 386], Gly-463 [auth 435] and Arg-481 [auth 453], suggesting that the ligands in these systems induced conformational changes in these specific regions.

### MM/GBSA BChE and occupancy of higrogen bonds

The binding energy calculations (ΔG_bind) and their energetic components for the 8U2-BChE, TSCZ4-BChE, and TSCZ6-BChE systems are summarized in Table 2. The 8U2-BChE system presented the most favorable binding energy, with a ΔG_bind_ of − 26.27 ± 3.89 kcal/mol, followed by TSCZ6-BChE (ΔG_bind_ of − 22.86 ± 3.02 kcal/mol) and TSCZ4-BChE (ΔG_bind_ of − 19.54 ± 2.73 kcal/mol). Notably, the 8U2-BChE system also obtained the lowest values for the van der Waals (E_vdW) and electrostatic (E_ele) interaction energies, with − 35.56 and − 8.43 kcal/mol, respectively, which contributes to its more excellent stability. Furthermore, nonpolar surface energy (G_SA_) values showed that 8U2-BChE had the most negative contribution (− 7.45 kcal/mol) compared to TSCZ6-BChE and TSCZ4-BChE.

**Table 4:** Predicted binding free energy (kcal/mol) and individual energy terms of the 8U2-BChE, TSCZ4-BChE and TSCZ6-BChE complexes

Term	8U2-BChE	TSCZ4-BChE	TSCZ6-BChE
E_vdW_	− 35.56	− 31.56	− 32.93
E_ele_	− 8.43	− 6.43	− 7.19
G_GB_	19.93	16.66	18.04
G_SA_	− 7.45	− 4.45	− 5.67
− TΔS	5.24	6.24	4.89
ΔG_bind;_	− 26.27 +/- 3.89	−19.54 +/- 2.73	− 22.86 +/− 3.02

**Fig. 18:**
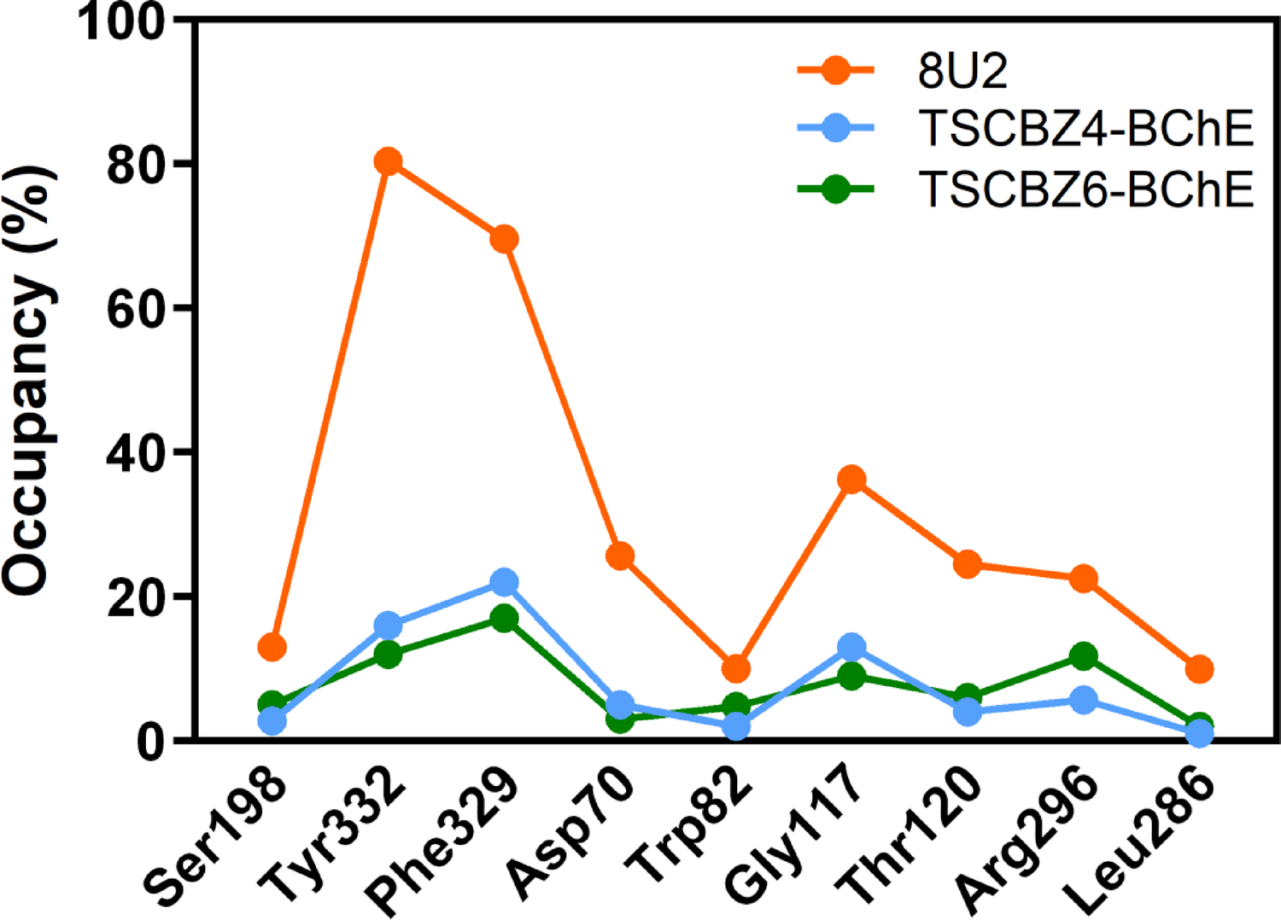
Hydrogen bond occupancy for 8U2-BChE, TSCZ4-BChE and TSCZ6-BChE complexes in %

The data presented in Fig. 18 shows the frequencies of hydrogen bonds between ligands and amino acid residues in the 8U2-BChE, TSCZ4-BChE, and TSCZ6-BChE systems. For the Tyr-332 residue, the 8U2-BChE system showed the highest binding frequency over time (80.39%), while the TSCZ4-BChE and TSCZ6-BChE systems had lower values, 16%, and 12%, respectively, but still maintaining links with this residue.

The Phe-329 residue also stood out, with the 8U2-BChE system presenting 69.61% against 22% and 17% in the TSCZ4-BChE and TSCZ6-BChE systems. For residue Asp-70, the frequency was 25.70% in the 8U2-BChE system, while TSCZ4-BChE and TSCZ6-BChE showed 5% and 3%, respectively. As for residue Gly-117, the 8U2-BChE system obtained 36.27%, while TSCZ4-BChE and TSCZ6-BChE recorded 13% and 9%, respectively.

## Discussion

### DFT analysis

The presence of the polar thiosemicarbazone and semicarbazone groups results in high calculated dipole moments (DM), Table S1, with the first group standing out as the most polar. The presence of the sp^2^ oxygen atom acts to decrease the net dipole moment in semicarbazones; in contrast, the presence of the sp^2^ sulfur atom in thiosemicarbazones acts to increase the net dipole moment in thiosemicarbazones. The results do not indicate a linear trend between the DM values and the electronegativity of the substituents on the benzene ring: TSCZ1 > TSCZ 3 > TSCZ2 > TSCZ4 > TSCZ6 > TSCZ5.

On the other hand, the results suggest that the polarizability of the thiophene and furan rings are similar because they both undergo resonance, which is not reflected in a significant effect on the energy gap results. Although the halogens have very different atomic radii, these substitutions had little significant effect on the energy gap values, in which it can be seen that the Br-substituted derivatives are more polarizable than the F-containing derivatives.

When comparing the two groups (thiosemicarbazones and semicarbazones), the sulfur suffers a character reversal, in which the sulfur in the thiosemicarbazone has a nucleophilic character; however, the sulfur in the thiophene has an electrophilic character; he is because, in thiophene, the sulfur participates in the ring resonance.

The results suggest that the S atom has greater nucleophilic character for the TSCZ1-3 derivatives and the N2 atom for the TSCZ4-6 derivatives; in comparison, the carbons of the benzene ring have greater electrophilic character for the TSCZ1-3 derivatives and the S atom for the TSCZ4-6 derivatives.

### Ecotoxicity

The behavior of solubility and log_Kow_ shows different patterns: while solubility increases with concentration, log_Kow_ decreases. This relationship becomes even more evident when the properties are analyzed in isolation (Table S7). Thus, the higher the log_Kow_ value, the lower the concentration/dose, which implies more significant toxicity of the contaminant to the organism. This is due to the lipophilic nature of these molecules, which indicate bioconcentration potential, as indicated by the log_Kow_ and BCF values. Furthermore, thiophene compounds can be bioconcentrated in marine organisms (Sinkkonen, 2001).

**Fig. 19:**
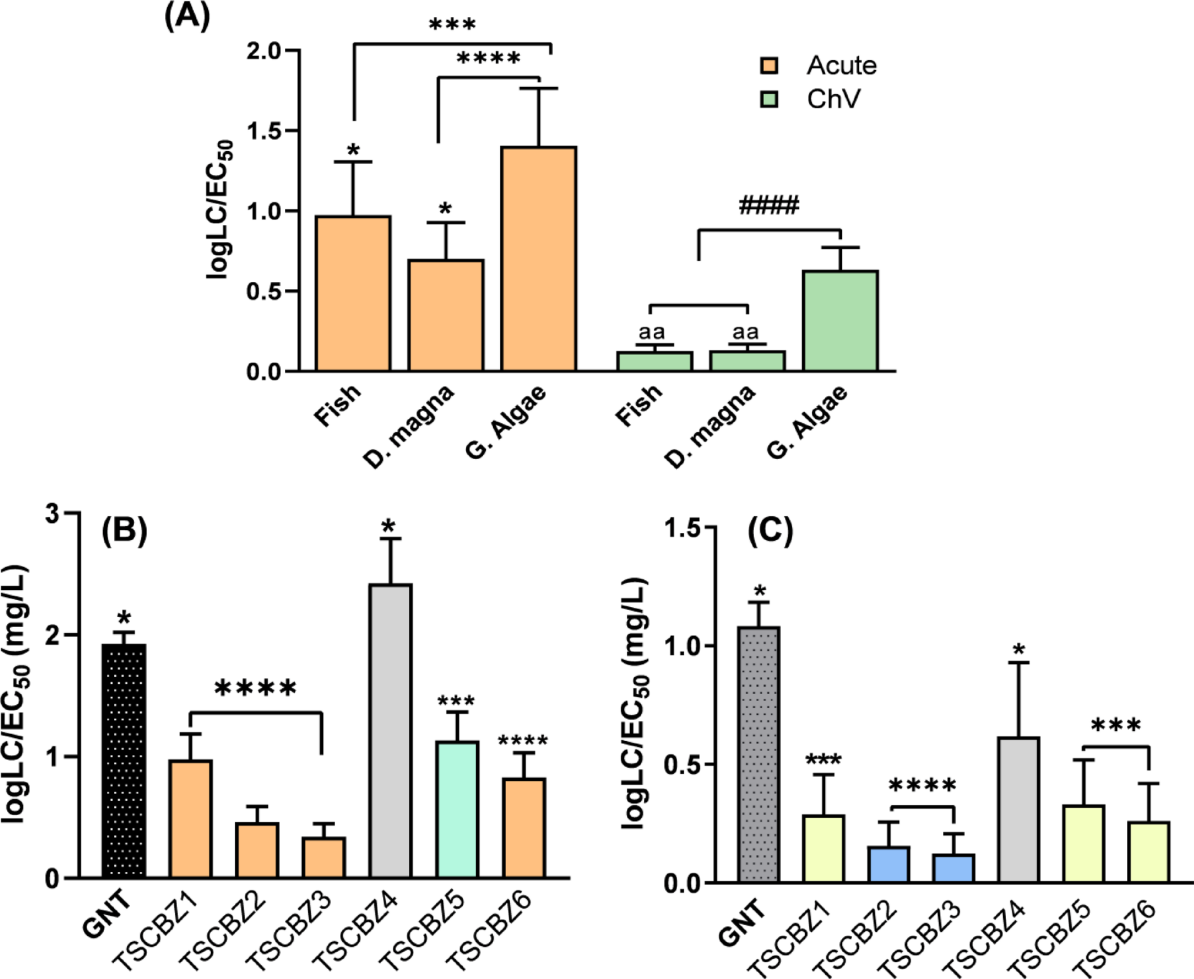
**A** Average acute (Fish LC_50_ 96 h, D. magna LC_50_ 48 h and G. algae EC_50_ 98 h) and chronic concentrations in aquatic organisms, **B** Acute concentrations of TSCBZ1-6 and **C** ChV concentrations of TSCBZ1-6. The values represent the mean ± standard error; ANOVA Two-way followed by Turkey (*p < 0.05, ***p < 0.001, ****p < 0.0001)

Furthermore, the average concentrations observed at acute levels show that the lowest concentrations are demonstrated by fish (96 h) and D. magna (48 h) with values ranging from 0.2 to 1.0 mg/L, in which latency will be the initial response. Another insight is the significant difference in concentrations in G. algae, due to the briefly high logKow values denoting a bioconcentrative effect throughout the population. This behavior is also evident in chronic concentrations, in which the neurological response is noticeable in the phylogenetic species fish and D. magna with concentrations below 0.1 mg/L, Fig. 19A.

Considering TSCBZ1-6, it was determined that these generally promote acetylcholinesterase modulation using galantamine (GNT - inhibitor via molecular docking) as a positive control, since TSCBZ1 (for –Br) semicarbazone portion and 4 (for –F) thiosemicarbazone portion (AChE molecular docking) exhibited the lowest and highest acute concentrations observed when compared to GNT for effect on the cholinergic system. However, TSCBZ4 and 6 (BChE molecular docking) established by the thiosemicarbazone fragments (para –F) have a similar effect at chronic concentrations (Jiang et al. 2023; Li et al. 2018).

Despite the recognized role of cholinesterase inhibition in neurotoxicity, ecotoxicological studies evaluating the effects of thiosemicarbazone derivatives on aquatic organisms remain scarce. Most previous research has focused on in vitro enzymatic assays or mammalian models, leaving the ecological relevance largely unexplored.

Nevertheless, evidence from existing studies indicates that cholinesterase inhibition can lead to significant behavioral alterations. For example, insecticide exposure in fish has been associated with changes in anxiety-related behaviors and locomotion due to altered AChE concentrations (Yang et al. 2023). Similarly, in vivo tests in adult rats showed that thiosemicarbazones possess anticonvulsant activity, reducing locomotor activity (Aly et al. 2010), while semicarbazone derivatives administered to mice exhibited sedative-hypnotic effects (Pandeya et al. 2002).

In this context, our analyses of TSCBZ derivatives 1–6 suggest potential biologically relevant outcomes. The high computational affinity observed for acetylcholinesterase (AChE) by TSCBZ1 and TSCBZ4 indicates a strong likelihood of enzymatic inhibition in aquatic organisms, potentially resulting in lethargy, convulsions, or immobilization, consistent with latency states predicted in fish and Daphnia magna. These findings are supported by previous studies, where xanthene‑thiosemicarbazones showed IC₅₀ values for AChE between 4.2 and 62 µM with selectivity over BChE (Naseem et al. 2024), and dimethoxyindole‑thiosemicarbazones exhibited dual inhibition with an IC₅₀ of 1.95 µM for BChE (Yıldız et al. 2022).

Moreover, our observation that sulfur atoms (TSCBZ1–3) and nitrogen atoms (TSCBZ4–6) constitute the most nucleophilic sites aligns with prior mechanistic studies, where heterocyclic S/N groups interact directly with catalytic residues of cholinesterases (Basri et al. 2023). From an ecological perspective, such inhibition may compromise motility, predation, and predator avoidance, providing a plausible mechanistic basis for the predicted toxicological effects, including latency, convulsions, and mortality observed in our models.

### Molecular Docking studies between TSCBZ1–6 and ache enzyme

**Fig. 20:**
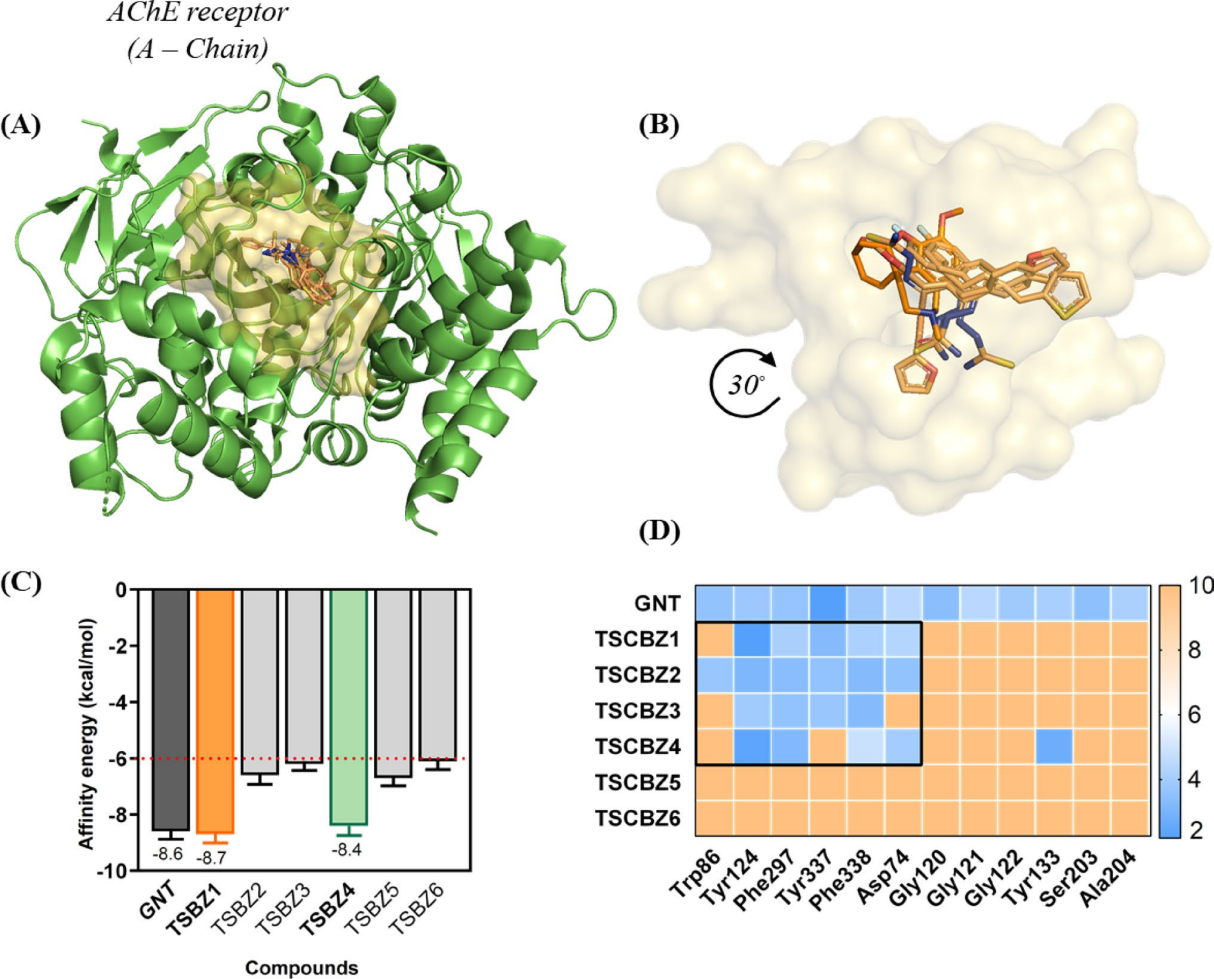
**A** Docking position of the GNT inhibitor and TSCBZ1-6 at the AChE enzyme, **B** Cavity of the GNT inhibitor and TSCBZ1-6, **C** Affinity energy and **D** Heatmap interactions (gradient of variation of low distances surfaces, azul to yellow color) and high distances surfaces (yellow to orange color)

The docking simulations showed a global localization (Fig. 20A) between the inhibitor GNT (galantamine) and TSBZ1-6, thus showing an analogous localization between GNT and SCBZ1-6 (Fig. 19B) in which selectivity to the AChE enzyme is delimited, given that GNT has established an affinity energy (∆G = − 8.6 kcal/mol^−1^), however, TSCBZ1 (∆G = − 8.7 kcal/mol^−1^) and TSCBZ1 (∆G = − 8.4 kcal/mol^−1^) were significant for molecular dynamics analysis. Furthermore, TSCBZ2 (∆G = − 6.6 kcal/mol^−1^), TSCBZ3 (∆G = − 6.2 kcal/mol^−1^), TSCBZ5 (∆G = − 6.7 kcal/mol^−1^) and TSCBZ6 (∆G = − 6.1 kcal/mol^−1^) denoted the affinity energies, respectively (Fig. 20C).

Based on the homologous localization between the GNT inhibitor (orange) and TCBZ1-6 (nude), an inhibitory effect can be established on the orthosteric site of the AChE human enzyme described with the overlap between the ligands studied. Given that TSCBZ1 shows the lowest RMSD (Root Mean Square Deviation) with a value of 1.120Å, followed by TSCBZ4 with a value of 1.355Å, as well as TSCBZ6 (1.637Å), GNT (1.672Å), TSCBZ5 (1.740Å), TSCBZ3 (1.913Å) and TSCBZ2 (1.995Å), respectively (Table S9).

The interactions of the amino acid residues and ligands (R-L) in question showed that TSCZB1 (Fig. 20D) has an inhibitory effect on the AChE enzyme through residues Phe297 (*d* = 4.18Å), Tyr337 (key to protein inhibition, *d* = 3.38Å), Phe338 (*d* = 4. 19Å) arranged under hydrophobic interactions and hydrogen bonds with residues Asp74 (*d* = 4.35Å) and Tyr124 (*d* = 2.19Å) with a range of interactions between 2–5Å denoting relevance in protein modulation between residues and ligands through the semicarbazone portion and the aliphatic chain associated with the furan.

However, TSCBZ4 also describes an inhibitory effect on AChE through residues Phe297 (*d* = 3.60Å) and Tyr337 (*d* = 3.24Å) identified by the presence of 2 (two) hydrophobic interactions, 2 (two) hydrogen bonds with residues Asp74 (*d* = 2.36Å) and Tyr124 (*d* = 2. 36Å), 1 (one) π-stacking (pararela) with Tyr338 (*d* = 4.86Å) and 1 (one) halogen bond with residue Ser203 (*d* = 2.74Å) (Fig. 20D) scoring a distance variation between 2.36 ~ 4.86Å describing the influence of the halo-substitution (*para*-F) and thiophene portion.

Molecular docking studies consistently reveal that thiosemicarbazone derivatives interact with acetylcholinesterase (AChE) through specific structural and electronic features, providing insights into their inhibitory potential and mechanistic basis. For instance, Kahvecioglu et al. (2024) demonstrated that pyrimidine-containing hydrazone and thiosemicarbazide derivatives engage both the catalytic and peripheral anionic sites of AChE via hydrogen bonding and π–π stacking, suggesting that heteroaromatic ring systems enhance binding affinity and stabilize enzyme–ligand complexes.

Uytun et al. (2022) reported that electron-donating substituents on thiosemicarbazone scaffolds strengthen interactions with catalytic residues, enabling dual inhibitory activity against AChE and monoamine oxidase B (MAO-B). Similarly, Khan et al. (2023) showed that para-substituted thiosemicarbazones establish critical hydrogen bonds with active site residues, with substitution patterns modulating both selectivity and binding energy, while Reynoso-García et al. (2025) emphasized that structural bioinformatics approaches can accurately predict binding hotspots and elucidate structure–activity relationships, facilitating rational inhibitor design.

Our analyses of TSCBZ derivatives 1–6 are in line with these findings. The high docking affinity observed for TSCBZ1 and TSCBZ4 toward AChE suggests a strong likelihood of enzymatic inhibition, consistent with the interaction patterns reported in previous studies. Notably, the sulfur atoms in TSCBZ1–3 and nitrogen atoms in TSCBZ4–6 were identified as the most nucleophilic centers, aligning with the mechanistic role of heteroatoms in stabilizing enzyme–ligand complexes through hydrogen bonding and electrostatic interactions. These observations support a structure–activity relationship in which electronic properties and heteroatom positioning are critical determinants of AChE inhibition.

From an ecological perspective, such inhibition may translate into behavioral and neurotoxic effects in aquatic organisms, including altered motility, predation, and predator avoidance, thereby providing a plausible mechanistic basis for the toxicological outcomes predicted in our models. By integrating computational docking, electronic analysis, and ecotoxicological considerations, our study extends the insights from previous work to environmentally relevant species, highlighting both the inhibitory potential and ecological implications of TSCBZ derivatives.

### Molecular docking between TSCBZ1–6 and BChE enzyme

**Fig. 21:**
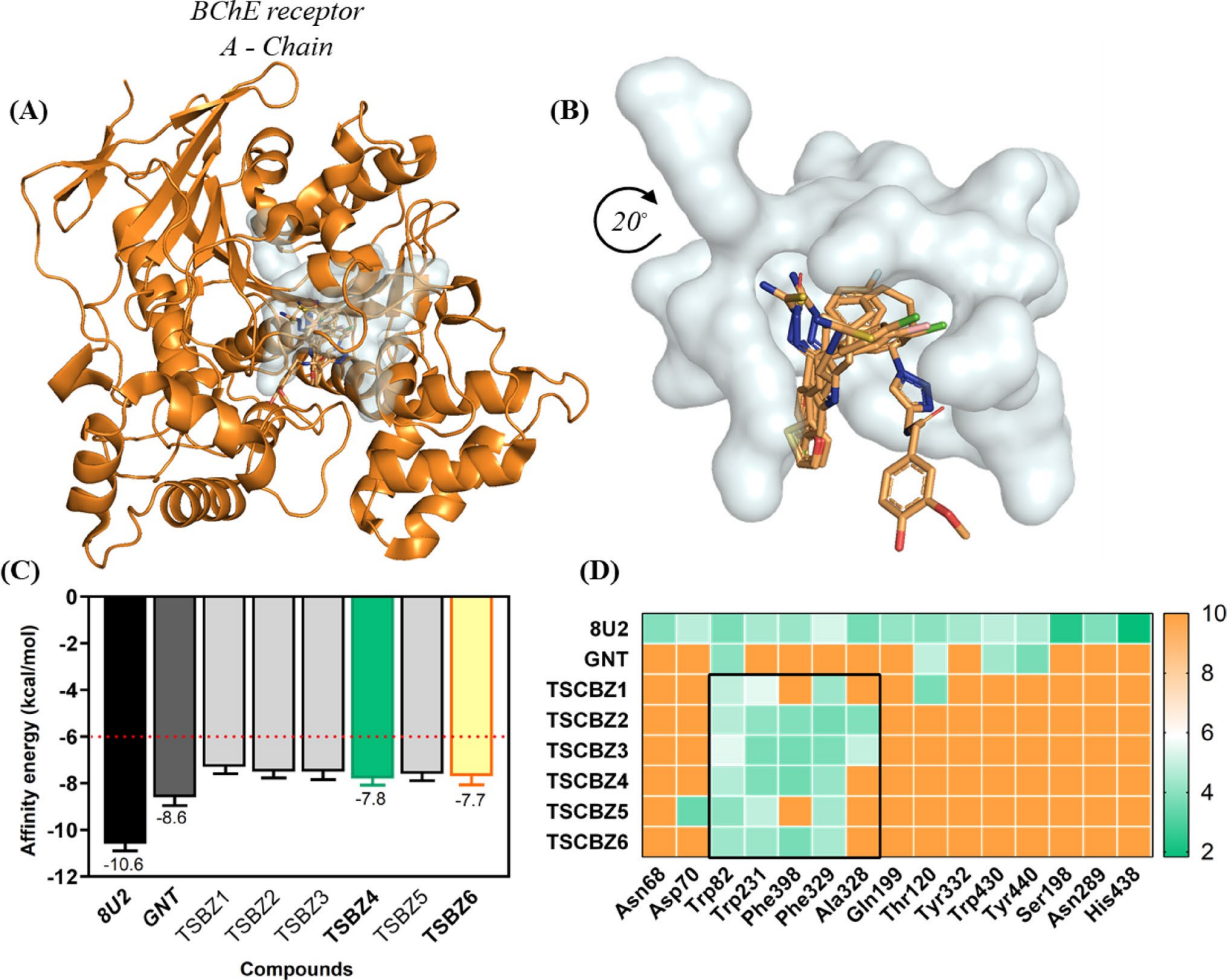
**A** Docking position of the 8U2 inhibitor and TSCBZ1-6 at the BChE enzyme, **B** Cavity of the 8U2 inhibitor and TSCBZ1-6, **C** Affinity energy and **D** Heatmap interactions (gradient of variation of low distances surfaces, verde to yellow color) and high distances surfaces (yellow to orange color)

Given the independent simulations between the inhibitor 8U2 (selective - BChE enzyme), GNT (selective - AChE enzyme) and TSCBZ1-6, with the general visualization (Fig. 21A) both molecules denote similar behavior when it comes to BChE inhibition, especially GNT and TSCBZ1-6, which are close due to the aliphatic chain and the quinolinimine group added by the heteroatom (Cl) (Fig. 21B).

Thus, the inhibitor 8U2 determines an energy value (affinity) of ∆G = − 10.6 kcal/mol^−1^, GNT (AChE inhibitor) with ∆G = -8.6 kcal/mol^−1^, however, TSCBZ1-6 oragnized in ascending order of affinity energy were: TSCBZ4 ∆G = − 7.8 kcal/mol^−1^ and TSCBZ6 ∆G = − 7.7 kcal/mol^−^^1^ (selected for molecular dynamics), TSCBZ5 ∆G = − 7.6 kcal/mol^−1^, TSCBZ3 ∆G = − 7.5 kcal/mol^−1^, TSCBZ2 ∆G = − 7.5 kcal/mol^−1^ and TSCBZ1∆G = − 7.3 kcal/mol^−1^ (Fig. 21C).

In addition, Root Mean Square Deviation (RMSD), a quantitative measure of overlap between the initial (0 simulations) and final (post-simulation) states of the ligands, showed that the inhibitor 8U2 (1.463Å), TSCBZ4 (1.538Å) and TSCBZ6 (1.570) provided lower RMSD values, i.e. a slight conformational variation upon complexation with BChE, while TSCBZ3 (1.644Å), GNT (1.672), TSCBZ2 (1.760Å), TSCBZ5 (1.871Å) and TSCBZ1 (1.913Å) provided values close to 2Å, respectively (Table S10).

Depending on the global localization between the 8U2 inhibitor, GNT and TSCBZ1-6, they proposed a modulatory effect on the BChE enzyme, given that the GNT inhibitor delimits this premise through the hydrophobic interactions enunciated by residues Trp82 (*d* = 4. 01Å) and Thr120 (*d* = 4.88Å), two (2) hydrogen bonds between residues Trp430 (*d* = 4.37Å) and Tyr440 (*d* = 3.70Å) (Fig. 21D). Whereas, TSCBZ4 assumes such similarity through four (4) hydrophobic residues which are Trp82 (*d* = 4.65Å), Trp231 (*d* = 3.78Å), Phe329 (*d* = 4.20Å), and Phe398 (*d* = 3.58Å), TSBZ6 commonly delimits interactions with residues Trp231 (hydrophobic, *d* = 4.28Å), Phe329 (hydrophobic, *d* = 4.47Å), Phe398 (hydrophobic, *d* = 3.70Å) and only one (1) π-stacking (parallel) with residue Trp82 (*d* = 4.32Å) (Fig. 21D).

Molecular docking studies on butyrylcholinesterase (BChE) have provided key insights into the structural features governing inhibitory potency of thiosemicarbazone derivatives. Al-Zahrani et al., (2023) demonstrated that thiosemicarbazones can interact with the BChE active site through hydrogen bonds and hydrophobic interactions, with heteroatoms such as sulfur and nitrogen playing a pivotal role in stabilizing the ligand–enzyme complex.

Similarly, Varma et al. (2023) reported that alkyl-substituted 4-methoxy benzaldehyde thiosemicarbazones exhibited strong BChE binding, indicating that electron-donating groups and aromatic substitutions enhance both affinity and selectivity, supporting their potential as multi-target directed ligands for neurodegenerative disorders. Essid et al. (2025) further highlighted that docking analyses, complemented by in vitro and molecular dynamics studies, reveal that proper positioning of nucleophilic centers facilitates interaction with catalytic and peripheral residues, directly influencing inhibitory efficacy and enzyme dynamics.

In the context of our TSCBZ derivatives 1–6, the docking results suggest a differential binding profile toward BChE compared with AChE. While TSCBZ1 and TSCBZ4 exhibited the highest affinity for AChE, the presence of nucleophilic sulfur and nitrogen atoms also favors interactions with BChE, potentially contributing to dual inhibition. These findings align with previous reports emphasizing that electronic properties, heteroatom positioning, and aromatic substitutions are critical determinants of cholinesterase inhibition. Considering the ecological and toxicological implications, BChE inhibition in aquatic organisms may further influence neuromuscular coordination and behavioral responses, reinforcing the relevance of these computational predictions for understanding potential environmental impacts of TSCBZ derivatives.

### Molecular dynamics

Molecular dynamics (MD) calculations play a key role in the detailed analysis of theoretical models. They allow an accurate assessment of the conformational changes, interactions, energies, and stability of a ligand-protein complex over time. These calculations provide detailed information about the interactions on an atomic scale, complementing the molecular docking study by providing a dynamic and more realistic view of the complex’s behavior (Cheatham et al. 1998).

Examining the trajectory files from the molecular dynamics (MD) simulations for the AChE protein made it possible to plot the RMSD results, providing a detailed view of conformational stability over time. To better understand the protein’s flexibility, simulations were conducted on the clean protein without residue or ligand. This enables a more precise comparison of how the presence of a ligand can affect the protein’s conformation, including possible denaturation or flexibility processes induced by interaction with the ligand (Gomes et al. 2023).

The results showed that, for the clean protein (without ligands), there were no major conformational changes after the thermal equilibrium stage, and the system showed good convergence of the trajectories. This represents a solid basis for comparisons with the complexes formed with the co-crystallized ligands (GNT), TSCZ4, and TSCZ1.

For the GNT-AChE complex, a more significant conformational variation was observed compared to the AChE-White system, suggesting that the co-crystallized ligand alters the conformation of the protein over time. In the case of the TSCZ4-AChE system, the RMSD results indicated that the TSCZ4 ligand also induces an increase in the protein’s conformational variation, indicating dynamic changes throughout the simulation. Finally, for the TSCZ1-AChE system, the RMSD results showed that the presence of the TSCZ1 ligand did not cause significant variations in the protein’s conformation, behaving in a similar way to the AChE-White system.

RMSF analysis makes it possible to quantify the structural fluctuations of the residues that make up the protein over time, offering a detailed view of the flexibility of the different molecule regions. This information is crucial for understanding the protein dynamics and identifying possible sites of biochemical interaction (Ghahremanian et al. 2022). With the trajectory files, it was possible to generate and analyze the RMSF results for all the systems studied, representing them in black, red, green, and blue, corresponding to the white-AChE, GNT-AChE, TSCZ4-AChE and TSCZ1-AChE systems. This analysis made it possible to identify the residues with the most significant fluctuations, establish a correlation with the RMSD results, and explain earlier conformational changes.

Current knowledge reveals that certain enzyme regions show more pronounced structural fluctuations, especially in the areas corresponding to the N-terminal and C-terminal residues and in loop regions on the surface of the protein (Lazim et al. 2020). This pattern was observed in all the systems studied, in which a significant fluctuation peak stood out in the N-terminal Glu-3 residue, with a variation of approximately 7.5 Å, and in the C-terminal residue, with a variation of around 5.2 Å.

Analysis of the RMSF results made it possible to compare the fluctuations over time between the different systems, revealing that the white-AChE and TSCZ1-AChE systems (represented by the black and blue colors) showed the slightest variations, with remarkable similarity between them, which indicates a low conformational variation of the protein. In contrast, the GNT-AChE and TSCZ4-AChE systems (represented by the red and green colors) showed more pronounced variations in specific residues, such as Phe-79, Val-269, Gly-335, His-386, Thr-435, and Ile-453.

These results suggest that the ligands interacted with the protein in such a way as to alter its conformation, affecting the residues mentioned. These findings corroborate the results obtained by RMSD analysis since the conformational changes observed were more evident. Thus, it can be concluded that the TSCBZ4 ligand induces conformational changes in AChE similar to those observed with the co-crystallized GNT ligand.

To improve the estimation of the binding free energy (ΔG_bind_), molecular dynamics (MD) simulations were carried out. These calculations require considerable processing power, mainly when calculating enthalpic and entropic contributions to the system. The simulations aimed to identify which simulated complexes had the most favorable ΔG_bind_ values, reflecting the best stability conditions. The results of these analyses are summarized in Table 3, which shows the variations in stability attributed to the specific interactions in each part of the complex.

Among the contributions observed in equation (Eq. 23), the term with the most incredible favorable intensity was the van der Waals energy value (E_vdW_). In contrast, the polar term (G_GB_) showed an unfavorable but decisive contribution to the calculation of ΔG_bind_. The results of the energy components for ΔG_bind_ in the GNT-AChE, TSCZ4-AChE, and TSCZ1-AChE systems indicate significant differences in the interactions of each ligand with the target protein. In the E_vdW_ term, stronger interactions were observed in the TSCZ4-AChE and TSCZ1-AChE systems compared to the GNT-AChE system (Table 4).

The TSCZ1-AChE system showed the most stable interaction, followed by TSCZ4-AChE, while GNT-AChE showed the least stable interactions. These results suggest that the TSCZ1-AChE and TSCZ4-AChE systems provide more favorable interactions than the GNT-AChE system.

The hydrogen bonds formed between the GNT-AChE, TSCZ4-AChE, and TSCZ1-AChE complexes. For this analysis, only interactions with occupancy values greater than 2% were considered since values below this threshold have no significant relevance for stabilizing the complex (Sharma et al. 2022).

The occupancy of hydrogen bonds based on molecular dynamics (MD) calculations, reflecting the frequency with which these interactions occur during the 200 ns simulation. The analysis focuses on the predominant interactions between the GNT inhibitor and the TSCZ4 and TSCZ1 ligands, considering only bonds with distances ≤ 3.5 Å. Interactions with low occupancy are considered less relevant to the stability of the complex and are, therefore, not detailed in this text (Kant et al. 2022; Zikri et al. 2020).

The residue occupancy results show marked differences in the interaction profiles between the GNT-AChE, TSCZ4-AChE, and TSCZ1-AChE systems. In the case of the Phe-295 residue, high occupancy was observed in both the GNT-AChE and TSCZ1-AChE systems, suggesting that this interaction is essential for the stability of the protein in these systems. On the other hand, in the TSCZ4-AChE system, Phe-295 occupancy was significantly lower.

Ser-125 and Trp-86 showed high occupancies in the TSCZ4-AChE and TSCZ1-AChE systems, with a sharper peak in TSCZ1-AChE. These data indicate that these residues play a fundamental role in stabilizing the interactions in the systems studied, suggesting that the TSCZ4 and TSCZ1 ligands induce conformations that reinforce these specific interactions.

In addition, the reduced occupancies for residues Leu-76, Ser-293, and Glu-292 in all systems suggest that these residues make a more negligible contribution to stabilizing the complexes.

On the other hand, residue Arg-296, which shows high occupancy in GNT-AChE but lower values in TSCZ4-AChE and TSCZ1-AChE, may be associated with a specific interaction with the GNT ligand. These differences indicate that the interaction profile of the TSCZ1-AChE system is more similar to that of GNT-AChE, especially on residues such as Phe-295.

The TSCZ4-AChE system, on the other hand, although showing less intense interactions in some key residues, maintains the same interaction site over time, as evidenced by the hydrogen bond frequencies.

Analysis of MD simulations of the BChE protein, without ligands or residues, allowed us to calculate RMSD values to evaluate the conformational behavior over time. The results were similar in the White-BChE systems (Run 1, Run 2, and Run 3). Initially, there was a variation of 1.55 Å until thermal equilibrium (1 ns), when the temperature was stabilized at 310 K.

Between 1 ns and 40 ns, Runs 2 and 3 showed a variation of 2.30 Å. In comparison, Run 1 had a minor variation of 1.57 Å. From 40 ns onwards, the three systems converged to a conformational variation of 2.25 Å, maintained until 140 ns. At this point, the variation increased to 3.10 Å but soon decreased and stabilized at about 2.20 Å until the end of the simulation (200 ns).

It was observed that, after the thermal equilibrium step, the BChE protein showed small conformational variations, indicating limited flexibility in its conformation over time. This behavior is a relevant result for comparison with the complexes formed with the co-crystallized ligand (8U2) and the ligands TSCZ4 and TSCZ6.

The comparison between the clean protein and the one complexed with the 8U2 inhibitor shows that the presence of the ligand influences the conformational behavior of the protein throughout the simulation. After equilibration, the complexed protein presents a more significant and more dynamic conformational variation, with peaks of up to 3.75 Å; this, the isolated protein stabilizes around 2.25 Å until approximately 140 ns, followed by an increase to 3.10 Å and, subsequently, stabilizes again at 2.20 Å until the end of the simulation.

These results suggest that the 8U2 inhibitor induces structural changes in BChE, promoting broader and more frequent fluctuations; this may reflect a conformational adaptation of the protein to accommodate the ligand, indicating a dynamic interaction between the protein and the inhibitor over time.

The MD results for the TSCZ4-BChE complex showed that, after thermal equilibrium, the system stabilized without showing conformational variations; this indicates that the ligand did not induce significant changes in the protein structure compared to the inhibitor 8U2. Similarly, the results for the TSCZ6-BChE complex also indicated that, after thermal equilibration, the system stabilized without relevant conformational variations, suggesting that the ligand did not cause significant structural changes in the BChE protein.

RMSF analyzes indicate that the presence of 8U2 and TSCZ4 ligands increases the flexibility of some regions of BChE, particularly in critical residues such as Ser-107 [auth 79], Pro-297 [auth 269], Gly-361 [auth 333], Arg-414 [auth 386], Gly-463 [auth 435] e Arg-481 [auth 453] (Viayna et al., 2020).

This greater flexibility may reflect a conformational adaptation of the protein to accommodate ligands, which is consistent with the RMSD results, which showed greater variations over time for these systems. On the other hand, the similarity in fluctuations observed between the White-BChE and TSCZ6-BChE systems, which presented lower RMSF values, suggests that the TSCZ6 ligand does not significantly alter the conformation of the protein, keeping it more stable.

This pattern of conformational behavior suggests that the TSCZ6 ligand may have an interaction that alters the conformation of BChE less, while the differences observed indicate how each ligand influences the structural flexibility of the protein, highlighting the regions of greatest fluctuation as possible sites of interaction. The inhibitor 8U2, which caused the largest fluctuations, especially at residue Glu-108 [auth 80], could induce more pronounced conformational changes, while the ligands TSCZ4 and TSCZ6 maintained the protein in a state closer to that observed in the absence of ligands.

The results indicated that the 8U2-BChE complex presents the most stable interaction, evidenced by the more negative binding energy (ΔG_bind_) compared to the TSCZ4-BChE and TSCZ6-BChE systems, which can be explained by the intensity of the Van Der Waals and electrostatics, which were more substantial in the 8U2-BChE system. These components suggest that the 8U2 ligand is better positioned and coupled to BChE, favoring proximity (E_vdW_) and electrostatic affinity interactions (Lambo et al. 2023).

The TSCZ4-BChE system, which presented the lowest binding energy, exhibited less favorable EvdW and Eele values, indicating a less efficient interaction between the ligand and the enzyme. Furthermore, the entropic term (− TΔS) also contributed negatively, suggesting that the TSCZ4 ligand has a less conformational restriction but without compensating for unfavorable interactions. These results show that the inhibitor 8U2 complexes more stably with BChE, while the ligands TSCZ4 and TSCZ6 form less stable complexes with the enzyme.

The hydrogen bonds between the GNT-AChE, TSCZ4-AChE and TSCZ1-AChE complexes, only considering interactions with occupancy values greater than 2%, as lower values are not relevant for the stabilization of the complex (Sharma et al. 2022).

The figure shows the occupancy calculated from molecular dynamics (MD) data, reflecting the frequency of hydrogen bonds between the ligands and the protein over 200 ns of simulation, and compares the predominant interactions of the 8U2 inhibitor with the TSCZ4 ligands and TSCZ6, considering bonds with distances ≤ 3.5 Å. Interactions with low occupancy are considered less relevant for the stability of the complex (Kant et al. 2022; Zikri et al. 2020).

The results indicate that the 8U2-BChE complex presents a distinct hydrogen bond occupancy profile, with a high percentage of occupancy at Tyr-332 and Phe-329; this highlights the importance of these bonds in enzymatic stability and function, providing advantages competitive with the ligands TSCZ4-BChE and TSCZ6-BChE. The higher binding frequency to the Asp-70 residue in the 8U2-BChE system suggests a crucial role in electrostatic interactions and enzyme dynamics.

Likewise, the high-frequency percentage for residue Gly-117 suggests that hydrogen bonds influence the flexibility of this region. These differences in occupancy percentages may directly affect the mechanisms of action and catalytic efficiency of the enzyme, as evidenced by the RMSD and RMSF results, which show different behaviors for the ligands compared to the inhibitor 8U2.

## Conclusion

DFT calculations showed that the TSCBZ1–6 derivatives adopt low-energy conformations with an orthogonal orientation of the benzene ring and exhibit predominantly hard and electrophilic character, enhanced by bromine substitution. Nucleophilicity is mainly associated with sulfur atoms in TSCBZ1–3 and with N2 in TSCBZ4–6.

Ligand-based toxicity predictions indicated high acute toxicity in aquatic organisms, with TSCBZ1, TSCBZ4, and TSCBZ6 also presenting potential chronic effects. Docking and molecular dynamics results highlighted TSCBZ4 as the most promising acetylcholinesterase inhibitor, displaying stable interactions and binding affinities comparable to or higher than galantamine. These findings contribute to understanding the structure–toxicity relationship and potential neurotoxic risks of thiosemicarbazone derivatives.

This study is limited to in silico analyses, and the toxicity and enzyme inhibition results are predictive in nature. Computational models may not fully represent biological complexity, environmental variability, or metabolic processes. Consequently, experimental toxicological and biochemical assays are required to validate the proposed inhibitory mechanisms and environmental safety profiles.

## Supplementary Information

Below is the link to the electronic supplementary material.

Supplementary Material 1

Supplementary Material 2

## Data Availability

All data was given in the supplement.

## Abbreviations

TSCZ: Thiosemicarbazones
AChE: Acetylcholinesterase
BChE: Butyrylcholinesterase
DFT: Density Functional Theory
IEF-PCM: Integral Equation Formalism - Polarizable Continuum Model
HOMO: Highest Occupied Molecular Orbital
LUMO: Lowest Unoccupied Molecular Orbital
ECOSAR: Ecological Structure Activity Relationship
NOEC: No-observed Effect Concentration
LOEC: Lowest Observed Effect Concentration
QSAR: Quantitative Structure-Activity Relationship
logKow: Octanol-water Partition Coefficient
LC50: Lethal Concentration
EC50: Effective Concentration
ChV: Chronic
BCF: Bioconcentration
BAF: Bioaccumulation
PBT: Persistence, Bioaccumulation, and Toxicity
CMR: Carcinogenicity, Mutagenicity, and Reprotoxicity
CNS MPO: Central Nervous System Multiparameter Optimization
BTS: Behavioral Toxicity Syndromes
TPSA: Polar Surface Area
pKa: Acidity/Basicity
MW: Molecular Weight
HBD: H-bond Donors
MMFF94: Merck Molecular Force Field
RMSD: Root Mean Square Deviation
MD: Molecular Dynamics
TIP3P: Transferable Intermolecular Potential with 3 points
RMSF: Root Mean Square Fluctuation
MM/GBSA: Molecular Mechanics of Generalized Born Surface Area
FMO: Frontier Molecular Orbitals
GCRs: Global Chemical Reactivity Descriptors
MHP: Maximum Hardness Principle
MEP: Molecular Electrostatic Potential
GNT: Galantamine
8U2: (2-｛1-[4-(12-amino-3-chloro-6,7,10,11-tetrahydro-7,11-methanocycloocta[b]quinolin-9-yl)butyl]-1H-1,2,3-triazol-4-yl｝-N-[4-hydroxy-3-mehoxybenzyl]acetamide)
