# Supplementary material for: Ligand and structure-based toxicological assessment of (thio)semicarbazones on cholinesterases
Source: J Comput Aided Mol Des. 2026 Jan 8;40(1):40. doi: 10.1007/s10822-025-00746-6 (PMC12779692; doi:10.1007/s10822-025-00746-6)
Supplement: Supplementary file 1 — Supplementary Material 1 [file 10822_2025_746_MOESM1_ESM.docx]

**Ligand and Structure-Based toxicological assessment of (Thio)semicarbazones on cholinesterases**

Damião Sampaio de Sousa^1^, Akenaton Onassis Cardoso Viana Gomes^1^, Caio Henrique Alexandre Roberto^2^, Anthony Barbosa Belarmino^3^, Francisco Rogênio da Silva Mendes^1^, Márcia Machado Marinho^3^, Pedro de Lima-Neto^4^, Gabrielle Silva Marinho^5^

^1^ Postgraduate Program in Natural Sciences, State University of Ceará, Fortaleza, CE, Brazil

^2^ Postgraduate Program in Biotechnology, State University of Ceará, Fortaleza, CE, Brazil

^3^ Natural Resources Bioprospecting and Monitoring Laboratory, Fortaleza, CE, Brazil

^4^ Federal University of Ceará, Department of Analytical and Physical Chemistry, Fortaleza, Ceará, Brazil

^4^ Faculty of Education, Sciences and Letters of Iguatu, CE, Brazil

* Corresponding author: [marcia.marinho@uece.br](mailto:marcia.marinho@uece.br)

| Damião S. de Sousa <https://orcid.org/0000-0003-2425-8228> | Gabrielle S. Marinho <https://orcid.org/0000-0001-8950-7497> |
| --- | --- |
| Akenaton O. C. V. Gomes <https://orcid.org/0000-0001-6515-0577> | Caio H. A. Roberto <https://orcid.org/0000-0001-7590-5830> |
| Fco. Rogênio S. Mendes <https://orcid.org/0000-0001-8357-6707> | Anthony B. Belarmino <https://orcid.org/0000-0002-5123-3248> |
| Márcia M. Marinho <https://orcid.org/0000-0002-7640-2220> | Pedro L. Neto <https://orcid.org/0000-0002-1613-4797> |

**Supplementary Material**


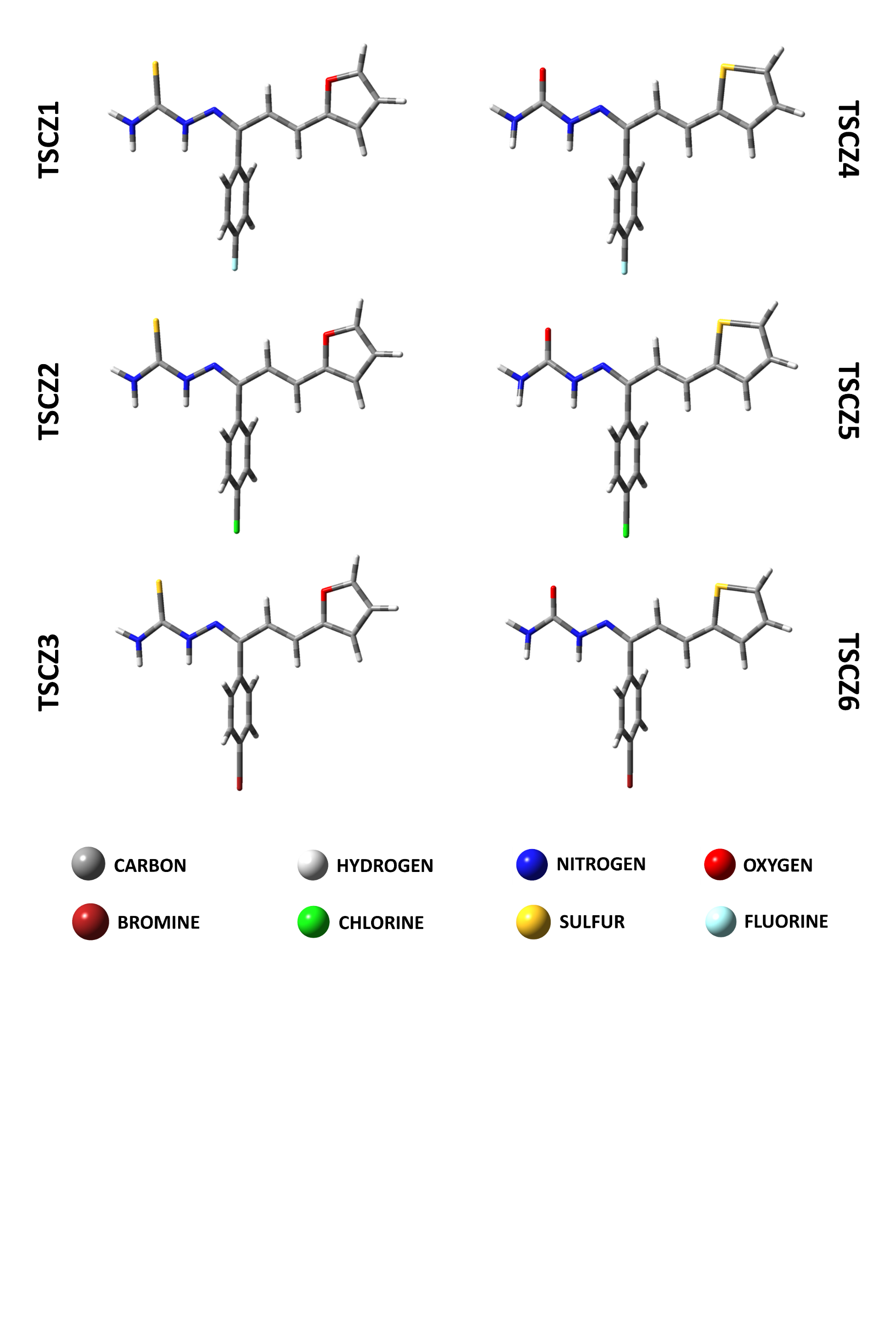
**Figure S1:** The optimized structure of TSCZ derivatives at DFT/B3LYP 6-311++G(d,p) calculated in Water

**Table S1.** Dipolar moment of TSCZ1-6 derivatives

| **Molecules** | **TSCZ1** | **TSCZ2** | **TSCZ3** | **TSCZ4** | **TSCZ5** | **TSCZ6** |
| --- | --- | --- | --- | --- | --- | --- |
| **µ (D)** | 9.293 | 9.142 | 9.248 | 6.786 | 6.623 | 6.731 |


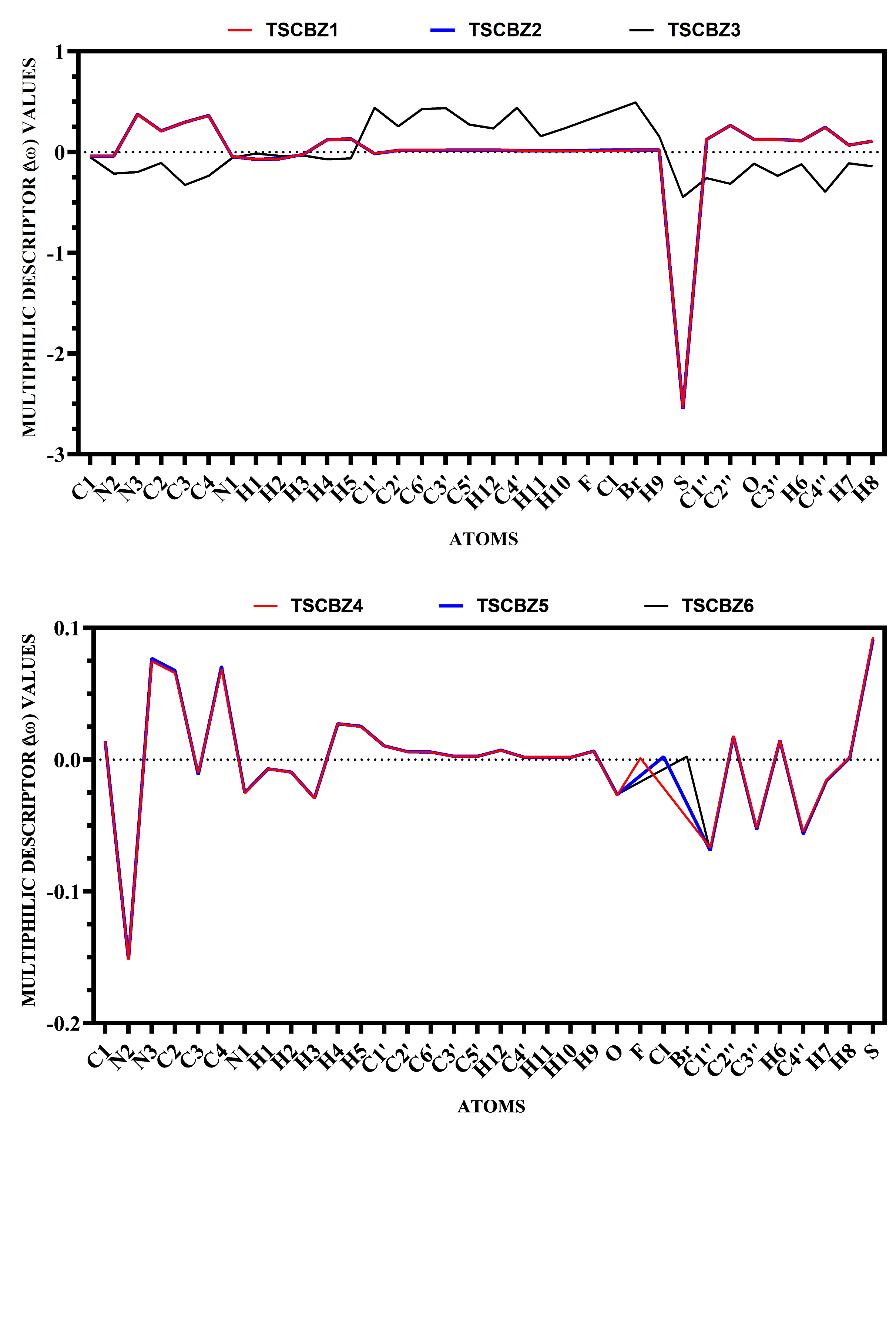
**Figure S2.** Multiphilic descriptor values for each atom of the TSCZ derivatives

**Table S2.** Global reactivity descriptors for TSCZ derivatives

| **Derivatives/Descriptors** | ***I*** | ***EA*** | ***χ*** | ***η*** | ***S*** | ***ω*** | ***ϵ*** |
| --- | --- | --- | --- | --- | --- | --- | --- |
| **TSCZ1** | 5.860 | 2.224 | 4.042 | 1.818 | 0.550 | 4.493 | 0222 |
| **TSCZ2** | 5.866 | 2.232 | 4.049 | 1.817 | 0.550 | 4.511 | 0.222 |
| **TSCZ3** | 5.867 | 2.233 | 4.050 | 1.817 | 0.550 | 4.514 | 0221 |
| **TSCZ4** | 5.844 | 2.128 | 3.986 | 1.858 | 0.538 | 4.276 | 0.234 |
| **TSCZ5** | 5.850 | 2.136 | 3.993 | 1.857 | 0.538 | 4.293 | 0.233 |
| **TSCZ6** | 5.851 | 2.137 | 3.994 | 1.857 | 0.538 | 4.295 | 0.233 |

**Table S3.** Condensed Fukui functions calculated by the Hirshfeld charge population for the TSCZ1-3

|  | **TSCBZ1** | | **TSCBZ2** | | **TSCBZ3** | |
| --- | --- | --- | --- | --- | --- | --- |
| **ATOM** | $f_{A}^{+}$ | $f_{A}^{-}$ | $f_{A}^{+}$ | $f_{A}^{-}$ | $f_{A}^{+}$ | $f_{A}^{-}$ |
| C1 | 0.039813 | 0.049044 | 0.039661 | 0.049065 | 0.005917 | 0.016863 |
| N2 | 0.016571 | 0.025593 | 0.016628 | 0.025549 | 0.004893 | 0.051919 |
| N3 | 0.092289 | 0.009064 | 0.092382 | 0.008909 | 0.022066 | 0.065828 |
| C2 | 0.067228 | 0.020633 | 0.067417 | 0.020608 | 0.006447 | 0.030395 |
| C3 | 0.068967 | 0.002969 | 0.068774 | 0.002963 | 0.006267 | 0.078425 |
| C4 | 0.096842 | 0.016395 | 0.096911 | 0.016353 | 0.010333 | 0.062645 |
| N1 | 0.031457 | 0.041302 | 0.031399 | 0.04132 | 0.006079 | 0.018861 |
| H1 | 0.014751 | 0.030857 | 0.014714 | 0.030848 | 0.006449 | 0.009422 |
| H2 | 0.0182 | 0.03297 | 0.018157 | 0.032943 | 0.004366 | 0.012771 |
| H3 | 0.012216 | 0.017419 | 0.012226 | 0.017414 | 0.010722 | 0.018414 |
| H4 | 0.031991 | 0.00483 | 0.031872 | 0.004797 | 0.01221 | 0.028235 |
| H5 | 0.034336 | 0.005463 | 0.034348 | 0.005441 | 0.010234 | 0.023893 |
| C1' | -0.00257 | 0.000555 | -0.00304 | 0.000253 | 0.092153 | -0.00552 |
| C2' | 0.008365 | 0.004492 | 0.008062 | 0.0043 | 0.061682 | 0.004929 |
| C6' | 0.008379 | 0.004533 | 0.008092 | 0.004319 | 0.099333 | 0.00495 |
| C3' | 0.009385 | 0.00478 | 0.008609 | 0.004335 | 0.102601 | 0.006087 |
| C5' | 0.009377 | 0.004806 | 0.008609 | 0.004347 | 0.066403 | 0.006085 |
| H12 | 0.008031 | 0.00337 | 0.007798 | 0.003238 | 0.056665 | 0.004796 |
| C4' | 0.009959 | 0.006129 | 0.008751 | 0.005404 | 0.103747 | 0.006147 |
| H11 | 0.005969 | 0.002997 | 0.00555 | 0.002771 | 0.038867 | 0.003851 |
| H10 | 0.005972 | 0.002991 | 0.005553 | 0.002769 | 0.055884 | 0.003853 |
| F | 0.00671 | 0.004189 | - | - | - | - |
| Cl | - | - | 0.012678 | 0.008312 | - | - |
| Br | - | - |  | - | 0.120148 | 0.010787 |
| H9 | 0.007942 | 0.003376 | 0.007756 | 0.003243 | 0.039117 | 0.004784 |
| S | 0.094463 | 0.659462 | 0.094256 | 0.658784 | 0.023685 | 0.122506 |
| C1'' | 0.030796 | 0.002866 | 0.030664 | 0.002864 | 0.002749 | 0.059823 |
| C2'' | 0.069106 | 0.010276 | 0.069043 | 0.010257 | 0.007569 | 0.077473 |
| O | 0.031121 | 0.003211 | 0.031045 | 0.003203 | 0.002948 | 0.028565 |
| C3'' | 0.032935 | 0.005201 | 0.032886 | 0.005195 | 0.003922 | 0.056275 |
| H6 | 0.029189 | 0.004051 | 0.029143 | 0.004041 | 0.004482 | 0.031399 |
| C4'' | 0.064072 | 0.00954 | 0.06399 | 0.009529 | 0.006845 | 0.094117 |
| H7 | 0.018098 | 0.00285 | 0.018072 | 0.002845 | 0.002242 | 0.02702 |
| H8 | 0.028043 | 0.003787 | 0.027988 | 0.003781 | 0.002977 | 0.034401 |

**Table S4**. Fukui functions calculated by the Hirshfeld charge population for the TSCZ4-6

|  | **TSCBZ4** | | **TSCBZ5** | | **TSCBZ6** | |
| --- | --- | --- | --- | --- | --- | --- |
| **ATOM** | $f_{A}^{+}$ | $f_{A}^{-}$ | $f_{A}^{+}$ | $f_{A}^{-}$ | $f_{A}^{+}$ | $f_{A}^{-}$ |
| C1 | 0.026141 | 0.022925 | 0.026113 | 0.022805 | 0.026106 | 0.022797 |
| N2 | 0.025506 | 0.061014 | 0.025599 | 0.060898 | 0.025612 | 0.060898 |
| N3 | 0.089788 | 0.072337 | 0.089932 | 0.072061 | 0.089964 | 0.072051 |
| C2 | 0.05208 | 0.036714 | 0.05235 | 0.036603 | 0.05237 | 0.036589 |
| C3 | 0.07942 | 0.08179 | 0.079211 | 0.08187 | 0.079203 | 0.081886 |
| C4 | 0.088454 | 0.072374 | 0.088629 | 0.072141 | 0.088658 | 0.07209 |
| N1 | 0.017511 | 0.023474 | 0.017522 | 0.023397 | 0.017523 | 0.023395 |
| H1 | 0.008887 | 0.010531 | 0.00889 | 0.010492 | 0.008887 | 0.010488 |
| H2 | 0.011286 | 0.013547 | 0.011286 | 0.013495 | 0.011286 | 0.013493 |
| H3 | 0.014649 | 0.021535 | 0.014693 | 0.021502 | 0.014688 | 0.02149 |
| H4 | 0.035833 | 0.029416 | 0.035706 | 0.029364 | 0.035716 | 0.029366 |
| H5 | 0.031882 | 0.026074 | 0.031912 | 0.026024 | 0.031896 | 0.025994 |
| C1' | -0.00311 | -0.00554 | -0.00352 | -0.00597 | -0.00352 | -0.00597 |
| C2' | 0.007296 | 0.005932 | 0.00705 | 0.005654 | 0.006989 | 0.005592 |
| C6' | 0.007317 | 0.006008 | 0.007071 | 0.00573 | 0.007013 | 0.005668 |
| C3' | 0.008184 | 0.007635 | 0.007526 | 0.006951 | 0.007426 | 0.006849 |
| C5' | 0.008174 | 0.007594 | 0.007516 | 0.006918 | 0.007412 | 0.006814 |
| H12 | 0.007302 | 0.005668 | 0.007124 | 0.005468 | 0.007076 | 0.005423 |
| C4' | 0.008891 | 0.008469 | 0.007855 | 0.007426 | 0.007243 | 0.006841 |
| H11 | 0.005157 | 0.004764 | 0.0048 | 0.004414 | 0.004688 | 0.00431 |
| H10 | 0.005169 | 0.004774 | 0.004815 | 0.004422 | 0.004705 | 0.004319 |
| H9 | 0.007082 | 0.005603 | 0.006908 | 0.005403 | 0.006863 | 0.005359 |
| O | 0.031076 | 0.037415 | 0.031056 | 0.03723 | 0.031049 | 0.037213 |
| F | 0.005946 | 0.005702 | - | - | - | - |
| Cl | - | - | 0.011244 | 0.010721 | - | - |
| Br | - | - | - | - | 0.01252 | 0.01197 |
| C1'' | 0.037938 | 0.053551 | 0.037699 | 0.053769 | 0.037677 | 0.053802 |
| C2'' | 0.072946 | 0.068709 | 0.072823 | 0.068719 | 0.07282 | 0.068715 |
| C3'' | 0.03813 | 0.050228 | 0.037977 | 0.050355 | 0.037965 | 0.050375 |
| H6 | 0.031698 | 0.028226 | 0.031615 | 0.02824 | 0.031606 | 0.028239 |
| C4'' | 0.074539 | 0.087337 | 0.074342 | 0.087495 | 0.074334 | 0.087517 |
| H7 | 0.02136 | 0.024967 | 0.021285 | 0.025011 | 0.021278 | 0.025017 |
| H8 | 0.033639 | 0.033172 | 0.033532 | 0.033217 | 0.033527 | 0.033223 |
| S | 0.109833 | 0.088053 | 0.10944 | 0.08817 | 0.109418 | 0.08819 |

**Table S5.** Calculated the Dual ($\Delta f$) and the multiphilic descriptors ($\Delta\omega$) for the TSCZ1-3

|  | **TSCBZ1** | | **TSCBZ2** | | **TSCBZ3** | |
| --- | --- | --- | --- | --- | --- | --- |
| **ATOM** | $\Delta f$ | $\Delta\omega$ | $\Delta f$ | $\Delta\omega$ | $\Delta f$ | $\Delta\omega$ |
| C1 | -0.00923 | -0.04148 | -0.0094 | -0.0424254 | -0.01095 | -0.04941 |
| N2 | -0.00902 | -0.04054 | -0.00892 | -0.0402461 | -0.04703 | -0.21226 |
| N3 | 0.083225 | 0.373957 | 0.083473 | 0.37657953 | -0.04376 | -0.19753 |
| C2 | 0.046596 | 0.209371 | 0.04681 | 0.21117635 | -0.02395 | -0.1081 |
| C3 | 0.065998 | 0.29655 | 0.065812 | 0.29690305 | -0.07216 | -0.32569 |
| C4 | 0.080447 | 0.361476 | 0.080558 | 0.36342819 | -0.05231 | -0.23612 |
| N1 | -0.00985 | -0.04424 | -0.00992 | -0.0447583 | -0.01278 | -0.0577 |
| H1 | -0.01611 | -0.07237 | -0.01613 | -0.0727861 | -0.00297 | -0.01342 |
| H2 | -0.01477 | -0.06636 | -0.01479 | -0.0667074 | -0.0084 | -0.03793 |
| H3 | -0.0052 | -0.02338 | -0.00519 | -0.0234038 | -0.00769 | -0.03472 |
| H4 | 0.027162 | 0.122046 | 0.027075 | 0.12214763 | -0.01603 | -0.07233 |
| H5 | 0.028873 | 0.129737 | 0.028907 | 0.13041042 | -0.01366 | -0.06165 |
| C1' | -0.00313 | -0.01405 | -0.00329 | -0.0148492 | 0.097672 | 0.440853 |
| C2' | 0.003873 | 0.017404 | 0.003762 | 0.01697136 | 0.056753 | 0.256161 |
| C6' | 0.003846 | 0.01728 | 0.003773 | 0.01702207 | 0.094383 | 0.42601 |
| C3' | 0.004605 | 0.020693 | 0.004273 | 0.0192784 | 0.096514 | 0.435626 |
| C5' | 0.004572 | 0.020542 | 0.004262 | 0.01922927 | 0.060318 | 0.272254 |
| H12 | 0.004662 | 0.020947 | 0.004561 | 0.02057493 | 0.05187 | 0.234121 |
| C4' | 0.00383 | 0.017208 | 0.003347 | 0.01510031 | 0.0976 | 0.44053 |
| H11 | 0.002971 | 0.01335 | 0.00278 | 0.01253991 | 0.035016 | 0.158049 |
| H10 | 0.002981 | 0.013394 | 0.002784 | 0.01255904 | 0.052031 | 0.234847 |
| F | 0.002521 | 0.011328 | - | - | - | - |
| Cl | - | - | 0.004366 | 0.01969796 | - | - |
| Br | - | - | - | - | 0.109361 | 0.493615 |
| H9 | 0.004567 | 0.02052 | 0.004513 | 0.02036055 | 0.034333 | 0.154966 |
| S | -0.565 | -2.53873 | -0.56453 | -2.5468065 | -0.09882 | -0.44604 |
| C1'' | 0.027931 | 0.125502 | 0.0278 | 0.12541527 | -0.05707 | -0.25761 |
| C2'' | 0.058829 | 0.264339 | 0.058786 | 0.26520772 | -0.0699 | -0.31552 |
| O | 0.027909 | 0.125406 | 0.027842 | 0.12560836 | -0.02562 | -0.11563 |
| C3'' | 0.027734 | 0.12462 | 0.027691 | 0.12492646 | -0.05235 | -0.2363 |
| H6 | 0.025138 | 0.112952 | 0.025102 | 0.11324566 | -0.02692 | -0.1215 |
| C4'' | 0.054531 | 0.245028 | 0.054462 | 0.24569784 | -0.08727 | -0.39391 |
| H7 | 0.015248 | 0.068515 | 0.015227 | 0.0686938 | -0.02478 | -0.11184 |
| H8 | 0.024255 | 0.108987 | 0.024207 | 0.10920882 | -0.03142 | -0.14184 |

**Table S6.** Calculated the Dual ($\Delta f$) and the multiphilic descriptors ($\Delta\omega$) for the TSCZ4-6

|  | **TSCBZ4** | | **TSCBZ5** | | **TSCBZ6** | |
| --- | --- | --- | --- | --- | --- | --- |
| **ATOM** | $\Delta f$ | $\Delta\omega$ | $\Delta f$ | $\Delta\omega$ | $\Delta f$ | $\Delta\omega$ |
| C1 | 0.003215 | 0.013748 | 0.003307 | 0.014198 | 0.003309 | 0.014212 |
| N2 | -0.03551 | -0.15182 | -0.0353 | -0.15154 | -0.03529 | -0.15156 |
| N3 | 0.017451 | 0.074612 | 0.017871 | 0.076717 | 0.017913 | 0.07694 |
| C2 | 0.015365 | 0.065697 | 0.015747 | 0.067603 | 0.015781 | 0.067781 |
| C3 | -0.00237 | -0.01014 | -0.00266 | -0.01142 | -0.00268 | -0.01152 |
| C4 | 0.016079 | 0.068749 | 0.016488 | 0.070783 | 0.016569 | 0.071164 |
| N1 | -0.00596 | -0.0255 | -0.00587 | -0.02522 | -0.00587 | -0.02522 |
| H1 | -0.00164 | -0.00703 | -0.0016 | -0.00688 | -0.0016 | -0.00688 |
| H2 | -0.00226 | -0.00967 | -0.00221 | -0.00948 | -0.00221 | -0.00948 |
| H3 | -0.00689 | -0.02944 | -0.00681 | -0.02923 | -0.0068 | -0.02922 |
| H4 | 0.006416 | 0.027434 | 0.006342 | 0.027224 | 0.006349 | 0.027271 |
| H5 | 0.005808 | 0.024835 | 0.005888 | 0.025275 | 0.005901 | 0.025347 |
| C1' | 0.002427 | 0.010377 | 0.002444 | 0.010491 | 0.002453 | 0.010535 |
| C2' | 0.001364 | 0.005831 | 0.001396 | 0.005994 | 0.001397 | 0.006002 |
| C6' | 0.001309 | 0.005597 | 0.001341 | 0.005757 | 0.001345 | 0.005776 |
| C3' | 0.000548 | 0.002344 | 0.000576 | 0.002471 | 0.000577 | 0.002479 |
| C5' | 0.00058 | 0.002478 | 0.000598 | 0.002566 | 0.000599 | 0.002572 |
| H12 | 0.001634 | 0.006987 | 0.001657 | 0.007112 | 0.001653 | 0.007101 |
| C4' | 0.000422 | 0.001806 | 0.000429 | 0.001842 | 0.000402 | 0.001726 |
| H11 | 0.000393 | 0.00168 | 0.000386 | 0.001657 | 0.000379 | 0.001626 |
| H10 | 0.000395 | 0.00169 | 0.000393 | 0.001688 | 0.000386 | 0.001658 |
| H9 | 0.00148 | 0.006326 | 0.001505 | 0.006462 | 0.001504 | 0.006461 |
| O | -0.00634 | -0.0271 | -0.00617 | -0.0265 | -0.00616 | -0.02647 |
| F | 0.000244 | 0.001042 | - | - | - | - |
| Cl | - | - | 0.000523 | 0.002244 | - | - |
| Br | - | - | - | - | 0.00055 | 0.002362 |
| C1'' | -0.01561 | -0.06676 | -0.01607 | -0.06899 | -0.01612 | -0.06926 |
| C2'' | 0.004237 | 0.018115 | 0.004104 | 0.017618 | 0.004105 | 0.017631 |
| C3'' | -0.0121 | -0.05173 | -0.01238 | -0.05314 | -0.01241 | -0.0533 |
| H6 | 0.003471 | 0.014842 | 0.003375 | 0.014491 | 0.003367 | 0.014462 |
| C4'' | -0.0128 | -0.05472 | -0.01315 | -0.05647 | -0.01318 | -0.05662 |
| H7 | -0.00361 | -0.01542 | -0.00373 | -0.016 | -0.00374 | -0.01606 |
| H8 | 0.000467 | 0.001998 | 0.000315 | 0.001354 | 0.000304 | 0.001304 |
| S | 0.02178 | 0.093124 | 0.02127 | 0.091311 | 0.021228 | 0.091175 |

**Table S7.** Ecotoxicity of thiosemicarbazones in aquatic organisms

|  |  |  |  |  |  |  |  |  |  | **Neutral organic** | | | | | |
| --- | --- | --- | --- | --- | --- | --- | --- | --- | --- | --- | --- | --- | --- | --- | --- |
|  |  |  |  | **Persistence (Days)** | | | **Ecotoxicological properties** | | | **Acute** **(mg/L)** | | | **Chronic** **(mg/L)** | | |
| **Comp** | **MW** | **log**  **kow** | **Solubility**  **(mg/L)** | **Water** | **Soil** | **Sediment** | **BCF**  **(L/Kg)** | **BAF**  **(L/Kg)** | **Half-life**  **(Days)** | **Fish**  **96h** | ***D. magna***  **48h** | **G. algae**  **96h** | **Fish** | ***D. magna*** | **G. algae** |
| **TSCZ1** | 289.33 | 4.695 | 1.385 | nP-7 | nP-41 | vP-227 | 499 | 501.3 | 1.325 | 0.903 | 0.657 | 1.370 | 0.118 | 0.128 | 0.624 |
| **TSCZ2** | 305.78 | 5.139 | 0.463 | nP-19 | nP-74 | nP-72 | 796 | 820.3 | 2.082 | 0.381 | 0.289 | **0.713*** | 0.052 | 0.063 | 0.356 |
| **TSCZ3** | 350.23 | 5.385 | 0.155 | nP-8 | nP-41 | P-127 | 764.4 | 801.9 | 1.988 | **0.263*** | **0.204*** | **0.553*** | 0.037 | 0.047 | **0.290*** |
| **TSCZ4** | 289.33 | 4.200 | 3.664 | nP-15 | nP-34 | vP-227 | 83.1 | 83.11 | 0.204 | 2.511 | 1.747 | 3.014 | 0.312 | 0.3 | 1.241 |
| **TSCZ5** | 305.78 | 4.644 | 1.227 | nP-22 | nP-74 | vP-227 | 132.4 | 132.4 | 0.321 | 1.059 | 0.768 | **1.570*** | 0.138 | 0.148 | 0.708 |
| **TSCZ6** | 350.23 | 4.890 | 0.411 | nP-5 | nP-41 | vP-227 | 127.3 | 127.4 | 0.306 | **0.730*** | **0.541*** | **1.216*** | 0.098 | 0.111 | **0.577*** |

**Notes:** MW – Molecular weight; log kow - Octanol-water partition coefficient; nP – Non persistent; P – Persistent; vP – Very persistent; BCF - Bioconcentration factor; BAF - Bioaccumulation factor; * - Concentration greater than solubility.

**Table S8.** Report know actives distribution and CNS MPO scores against the AChE of TSCBZ1-6 derivatives

| **Compd** | **log_Kow_** | **logD** | **MW** | **TPSA (Å)** | **HBD** | **pKa** | **CNS MPO** | **Actives 3D** |
| --- | --- | --- | --- | --- | --- | --- | --- | --- |
| **TSBZ1** | 4.695 | 3.07 | 289.33 | 63.55 | 3 | 2.01 | **4.68** | 53.0 |
| **TSBZ2** | 5.139 | 3.53 | 305.78 | 63.55 | 3 | 1.96 | 4.22 | 54.0 |
| **TSBZ3** | 5.385 | 3.70 | 350.23 | 63.55 | 3 | 2.34 | 4.05 | 57.0 |
| **TSBZ4** | 4.200 | 3.04 | 289.33 | 67.48 | 3 | 1.65 | **4.71** | 177.0 |
| **TSBZ5** | 4.644 | 3.50 | 305.78 | 67.48 | 3 | 1.61 | 4.25 | 184.0 |
| **TSBZ6** | 4.890 | 3.66 | 350.23 | 67.48 | 3 | 1.99 | **4.09** | 176.0 |

**Notes:** MW – Molecular weight; log k_ow_ - Octanol-water partition coefficient, TPSA - polar surface area, logD - pH buffer lipophilicity, HBD - H-bond donors, pKa - acidity/basicity and CNS MPO – Central Nervous System Multiparameter Optimization.

**Table 9.** Data on ligand-receptor (L-R) interaction in the redocking process of the ***GNT*** and molecular docking of the thiosemicarbazones (SCBZ1-6), via ***AChE***. These include: RMSD statistical adjustment, affinity energy, interaction amino acid residues, ligand-receptor distance (L-R) and types of interaction

| Compd | Energy | RMSD | Residue | Dis(L-R) (Å) | Inter. type |
| --- | --- | --- | --- | --- | --- |
| GNT | -8.6 | 1.672 | Trp86A | 3.86 | Hydrophobic |
|  |  |  | Trp86A | 4.47 | Hydrophobic |
|  |  |  | Trp86A | 3.60 | Hydrophobic |
|  |  |  | Trp86A | 3.79 | Hydrophobic |
|  |  |  | Tyr124A | 3.80 | Hydrophobic |
|  |  |  | Phe297A | 4.31 | Hydrophobic |
|  |  |  | Phe297A | 3.65 | Hydrophobic |
|  |  |  | Tyr337A | 3.97 | Hydrophobic |
|  |  |  | Phe338A | 3.87 | Hydrophobic |
|  |  |  | Asp74A | 4.48 | H-bond |
|  |  |  | Gly120A | 3.86 | H-bond |
|  |  |  | Gly120A | 3.41 | H-bond |
|  |  |  | Gly121A | 4.48 | H-bond |
|  |  |  | Gly122A | 3.88 | H-bond |
|  |  |  | Tyr337A | 2.19 | H-bond |
|  |  |  | Tyr133A | 4.11 | H-bond |
|  |  |  | Ser203A | 3.48 | H-bond |
|  |  |  | Ala204A | 4.15 | H-bond |
| TSCZ1 | -8.7 | 1.120 | Trp286A | 3.84 | Hydrophobic |
|  |  |  | Val294A | 4.07 | Hydrophobic |
|  |  |  | Phe297A | 4.12 | Hydrophobic |
|  |  |  | **Phe297A** | 4.18 | Hydrophobic |
|  |  |  | Phe297A | 4.62 | Hydrophobic |
|  |  |  | Tyr337A | 3.61 | Hydrophobic |
|  |  |  | **Tyr337A** | 3.28 | Hydrophobic |
|  |  |  | **Phe338A** | 4.19 | Hydrophobic |
|  |  |  | Phe338A | 4.53 | Hydrophobic |
|  |  |  | Tyr341A | 3.84 | Hydrophobic |
|  |  |  | Tyr341A | 4.42 | Hydrophobic |
|  |  |  | **Asp74A** | 4.35 | H-bond |
|  |  |  | Asp74A | 4.59 | H-bond |
|  |  |  | **Tyr124A** | 2.19 | H-bond |
|  |  |  | Ser125A | 3.13 | H-bond |
|  |  |  | Ser125A | 3.46 | H-bond |
|  |  |  | Phe295A | 3.32 | H-bond |
|  |  |  | Glu202A | 3.78 | Halogen bond |
| TSCBZ2 | -6.6 | 1.995 | Asp74A | 4.84 | Hydrophobic |
|  |  |  | **Trp86A** | 3.75 | Hydrophobic |
|  |  |  | Trp86A | 4.16 | Hydrophobic |
|  |  |  | Asn87A | 4.76 | Hydrophobic |
|  |  |  | Tyr124A | 4.35 | Hydrophobic |
|  |  |  | **Phe297A** | 3.35 | Hydrophobic |
|  |  |  | **Tyr337A** | 3.58 | Hydrophobic |
|  |  |  | **Phe338A** | 3.28 | Hydrophobic |
|  |  |  | Tyr341A | 4.34 | Hydrophobic |
|  |  |  | Tyr341A | 4.82 | Hydrophobic |
|  |  |  | **Asp74A** | 3.60 | H-bond |
|  |  |  | **Tyr124A** | 3.13 | H-bond |
|  |  |  | His447A | 4.20 | H-bond |
|  |  |  | His447A | 2.47 | H-bond |
| TSBZ3 | -6.2 | 1.913 | **Tyr124A** | 3.91 | Hydrophobic |
|  |  |  | Trp286A | 3.80 | Hydrophobic |
|  |  |  | **Phe297A** | 3.59 | Hydrophobic |
|  |  |  | Tyr337A | 3.01 | Hydrophobic |
|  |  |  | Tyr337A | 3.72 | Hydrophobic |
|  |  |  | **Phe338A** | 3.28 | Hydrophobic |
|  |  |  | Tyr341A | 3.44 | Hydrophobic |
|  |  |  | Tyr72A | 3.93 | H-bond |
|  |  |  | Tyr124A | 2.42 | H-bond |
|  |  |  | **Tyr337A** | 2.73 | H-bond |
|  |  |  | Tyr341A | 3.73 | H-bond |
|  |  |  | Tyr341A | 4.45 | π-stacking |
| TSBZ4 | -8.4 | 1.355 | Tyr124A | 4.67 | Hydrophobic |
|  |  |  | Tyr124A | 4.10 | Hydrophobic |
|  |  |  | Trp286A | 4.20 | Hydrophobic |
|  |  |  | Trp286A | 4.34 | Hydrophobic |
|  |  |  | Val294A | 4.35 | Hydrophobic |
|  |  |  | Phe297A | 4.19 | Hydrophobic |
|  |  |  | **Phe297A** | 3.60 | Hydrophobic |
|  |  |  | Phe297A | 3.73 | Hydrophobic |
|  |  |  | Tyr337A | 3.24 | Hydrophobic |
|  |  |  | Tyr341A | 3.70 | Hydrophobic |
|  |  |  | Tyr341A | 3.95 | Hydrophobic |
|  |  |  | **Asp74A** | 3.99 | H-bond |
|  |  |  | **Tyr124A** | 2.36 | H-bond |
|  |  |  | Tyr124A | 3.48 | H-bond |
|  |  |  | Tyr341A | 3.84 | H-bond |
|  |  |  | **Phe338A** | 4.86 | π-stacking |
|  |  |  | **Ser203A** | 2.74 | Halogen bond |
| TSBZ5 | -6.7 | 1.740 | Val330A | 4.97 | Hydrophobic |
|  |  |  | Val429A | 4.79 | Hydrophobic |
|  |  |  | Val429A | 3.80 | Hydrophobic |
|  |  |  | Tyr510A | 4.77 | Hydrophobic |
|  |  |  | Leu524A | 4.63 | Hydrophobic |
|  |  |  | Arg525A | 3.91 | Hydrophobic |
|  |  |  | Arg525A | 3.45 | Hydrophobic |
|  |  |  | Gln527A | 3.63 | Hydrophobic |
|  |  |  | Ala528A | 4.95 | Hydrophobic |
|  |  |  | Tyr510A | 3.15 | H-bond |
|  |  |  | Tyr510A | 1.87 | H-bond |
|  |  |  | Arg521A | 4.00 | H-bond |
|  |  |  | Val331A | 3.49 | Halogen bond |
| TSBZ6 | -6.1 | 1.637 | Val429A | 3.94 | Hydrophobic |
|  |  |  | Tyr510A | 4.95 | Hydrophobic |
|  |  |  | Tyr510A | 4.62 | Hydrophobic |
|  |  |  | Leu524A | 4.69 | Hydrophobic |
|  |  |  | Arg525A | 3.97 | Hydrophobic |
|  |  |  | Arg525A | 3.61 | Hydrophobic |
|  |  |  | Gln527A | 3.67 | Hydrophobic |
|  |  |  | Gln527A | 4.35 | Hydrophobic |
|  |  |  | Tyr510A | 3.18 | H-bond |
|  |  |  | Tyr510A | 2.08 | H-bond |
|  |  |  | Arg521A | 3.89 | H-bond |

**Table 10.** Data on ligand-receptor (L-R) interaction in the redocking process of the ***8U2*** and molecular docking of the thiosemicarbazones (TSCBZ1-6), via ***BChE***. These include: RMSD statistical adjustment, affinity energy (ΔG), interaction amino acid residues, ligand-receptor distance (L-R) and types of interaction

| Compd | Energy | RMSD | Residue | Dis(L-R) (Å) | Inter. type |
| --- | --- | --- | --- | --- | --- |
| 8U2 | -10.6 | 1.463 | Asn68A | 3.90 | Hydrophobic |
|  |  |  | Asp70A | 4.80 | Hydrophobic |
|  |  |  | Trp82A | 4.01 | Hydrophobic |
|  |  |  | Trp82A | 3.75 | Hydrophobic |
|  |  |  | Trp82A | 4.16 | Hydrophobic |
|  |  |  | Gln199A | 4.16 | Hydrophobic |
|  |  |  | Thr120A | 4.03 | Hydrophobic |
|  |  |  | Trp231A | 4.52 | Hydrophobic |
|  |  |  | Ala328A | 3.66 | Hydrophobic |
|  |  |  | Tyr332A | 4.43 | Hydrophobic |
|  |  |  | Phe398A | 4.25 | Hydrophobic |
|  |  |  | Phe398A | 3.69 | Hydrophobic |
|  |  |  | Trp430A | 4.83 | Hydrophobic |
|  |  |  | Tyr440A | 4.53 | Hydrophobic |
|  |  |  | Ser198A | 2.34 | H-bond |
|  |  |  | Asn289A | 3.80 | H-bond |
|  |  |  | His438A | 1.83 | H-bond |
|  |  |  | Phe329A | 5.23 | π-stacking |
| GNT | -8.6 | 1.672 | **Trp82A** | 4.01 | Hydrophobic |
|  |  |  | **Thr120A** | 4.88 | Hydrophobic |
|  |  |  | Gly115A | 4.27 | H-bond |
|  |  |  | Gly116A | 2.98 | H-bond |
|  |  |  | **Trp430A** | 4.37 | H-bond |
|  |  |  | **Tyr440A** | 3.70 | H-bond |
|  |  |  | Trp82A | 3.96 | π-stacking |
|  |  |  | Trp82A | 3.98 | π-stacking |
| TSCBZ1 | -7.3 | 1.913 | **Trp82A** | 4.88 | Hydrophobic |
|  |  |  | Leu286A | 4.74 | Hydrophobic |
|  |  |  | **Phe329A** | 4.32 | Hydrophobic |
|  |  |  | Phe398A | 4.51 | Hydrophobic |
|  |  |  | Trp82A | 3.50 | H-bond |
|  |  |  | Gly116A | 3.62 | H-bond |
|  |  |  | Gly117A | 3.84 | H-bond |
|  |  |  | **Thr120A** | 3.72 | H-bond |
|  |  |  | Trp82A | 4.10 | π-stacking |
|  |  |  | Trp82A | 4.52 | π-stacking |
|  |  |  | **Trp231A** | 5.49 | π-stacking |
|  |  |  | Phe329A | 5.00 | π-stacking |
| TSCBZ2 | -7.5 | 1.760 | **Trp82A** | 4.71 | Hydrophobic |
|  |  |  | **Trp231A** | 4.08 | Hydrophobic |
|  |  |  | Leu286A | 4.54 | Hydrophobic |
|  |  |  | Leu286A | 4.18 | Hydrophobic |
|  |  |  | Val288A | 4.06 | Hydrophobic |
|  |  |  | **Ala328A** | 3.85 | Hydrophobic |
|  |  |  | **Phe329A** | 3.64 | Hydrophobic |
|  |  |  | Phe329A | 3.65 | Hydrophobic |
|  |  |  | **Phe398A** | 3.86 | Hydrophobic |
|  |  |  | Tyr128A | 4.11 | H-bond |
|  |  |  | Glu197A | 3.50 | H-bond |
|  |  |  | Glu197A | 2.07 | H-bond |
|  |  |  | Ser198A | 2.72 | H-bond |
|  |  |  | Trp231A | 4.87 | π-stacking |
|  |  |  | Trp231A | 4.79 | π-stacking |
| TSCBZ3 | -7.5 | 1.644 | **Trp231A** | 3.74 | Hydrophobic |
|  |  |  | Leu286A | 3.93 | Hydrophobic |
|  |  |  | Val288A | 4.40 | Hydrophobic |
|  |  |  | **Ala328A** | 4.93 | Hydrophobic |
|  |  |  | Phe329A | 4.66 | Hydrophobic |
|  |  |  | **Phe329A** | 3.79 | Hydrophobic |
|  |  |  | Phe329A | 4.06 | Hydrophobic |
|  |  |  | **Phe398A** | 3.63 | Hydrophobic |
|  |  |  | Gly115A | 2.96 | H-bond |
|  |  |  | Gly117A | 3.94 | H-bond |
|  |  |  | Tyr128A | 2.49 | H-bond |
|  |  |  | Tyr128A | 2.07 | H-bond |
|  |  |  | Glu197A | 4.00 | H-bond |
|  |  |  | Ser198A | 2.43 | H-bond |
|  |  |  | **Trp82A** | 5.45 | π-stacking |
| TSCBZ4 | -7.8 | 1.538 | **Trp82A** | 4.65 | Hydrophobic |
|  |  |  | **Trp231A** | 3.78 | Hydrophobic |
|  |  |  | Trp231A | 4.44 | Hydrophobic |
|  |  |  | Leu286A | 3.89 | Hydrophobic |
|  |  |  | **Phe329A** | 4.20 | Hydrophobic |
|  |  |  | **Phe398A** | 3.58 | Hydrophobic |
|  |  |  | Gly115A | 4.20 | H-bond |
|  |  |  | Gly117A | 3.81 | H-bond |
|  |  |  | Tyr128A | 3.83 | H-bond |
|  |  |  | Ser198A | 3.36 | H-bond |
|  |  |  | Phe329A | 5.05 | π-stacking |
| TSCBZ5 | -7.6 | 1.871 | Trp82A | 4.15 | Hydrophobic |
|  |  |  | **Trp82A** | 4.06 | Hydrophobic |
|  |  |  | Leu286A | 3.74 | Hydrophobic |
|  |  |  | Val288A | 4.40 | Hydrophobic |
|  |  |  | **Phe329A** | 4.44 | Hydrophobic |
|  |  |  | Glu197A | 3.22 | H-bond |
|  |  |  | Gly117A | 3.76 | H-bond |
|  |  |  | Ser198A | 3.57 | H-bond |
|  |  |  | Trp231A | 5.02 | π-stacking |
|  |  |  | **Trp231A** | 4.89 | π-stacking |
|  |  |  | Phe329A | 5.44 | π-stacking |
|  |  |  | **Asp70A** | 3.46 | Halogen bond |
|  |  |  | Ser79A | 3.56 | Halogen bond |
| TSCBZ6 | -7.7 | 1.570 | Trp82A | 4.35 | Hydrophobic |
|  |  |  | Trp82A | 4.81 | Hydrophobic |
|  |  |  | Trp82A | 4.96 | Hydrophobic |
|  |  |  | **Trp231A** | 4.28 | Hydrophobic |
|  |  |  | Trp231A | 4.88 | Hydrophobic |
|  |  |  | Leu286A | 4.18 | Hydrophobic |
|  |  |  | **Phe329A** | 4.47 | Hydrophobic |
|  |  |  | **Phe398A** | 3.70 | Hydrophobic |
|  |  |  | Gly116A | 4.02 | H-bond |
|  |  |  | Gly115A | 2.87 | H-bond |
|  |  |  | **Trp82A** | 4.32 | π-stacking |
|  |  |  | Phe329A | 4.89 | π-stacking |
