# Supplementary material for: Ligand and structure-based toxicological assessment of (thio)semicarbazones on cholinesterases
Source: J Comput Aided Mol Des. 2026 Jan 8;40(1):40. doi: 10.1007/s10822-025-00746-6 (PMC12779692; doi:10.1007/s10822-025-00746-6)
Supplement: Supplementary file 2 — Supplementary Material 2 [file 10822_2025_746_MOESM2_ESM.docx]

**Conclusions** — a very brief summary

The TSCBZ1–6 derivatives exhibited stable geometries and electronic properties dominated by the (thio)semicarbazone nucleus, little affected by aromatic substitution. The presence of Br increased the electrophilic character, while the nucleophilic centers varied between S (TSCBZ1–3) and N_2_ (TSCBZ4–6).

Toxicological predictions indicated that TSCBZ1, 4, and 6 can induce acute and chronic effects in aquatic organisms, while the others showed narcotic potential.

Docking and molecular dynamics results pointed to TSCBZ4 as the most promising compound, showing greater affinity and stability in the complex with AChE than the standard inhibitor. For BChE, TSCBZ4 and 6 showed moderate affinity, without inducing relevant structural changes.

Taken together, the data show that electronic and structural properties determine the toxicological profile and the selectivity of cholinesterase inhibition, highlighting TSCBZ4 as a potential selective inhibitor of AChE.
